# RECC: A Red/ET–CRISPR/Cas9-based system enabling genome mining of marine *Pseudoalteromonas* for novel natural products

**DOI:** 10.1016/j.synbio.2025.12.015

**Published:** 2026-01-10

**Authors:** Zong-jie Wang, Haibo Zhou, Youming Zhang, Fu Yan, Liujie Huo, Xiaotong Wang

**Affiliations:** a Helmholtz International Laboratory, State Key Laboratory of Microbial Technology, Shandong University, Qingdao, 266237, China; b Hunan Provincial Key Laboratory of Microbial Molecular Biology, College of Life Science, Hunan Normal University, Changsha, 410081, China

**Keywords:** *Pseudoalteromonas*, Genome mining, Natural products, Recombinases

## Abstract

Marine microorganisms possess vast biosynthetic potential, yet most of their biosynthetic gene clusters (BGCs) remain transcriptionally silent under laboratory conditions. Genetic intractability has been a major barrier to activating these cryptic pathways. Here, we present RECC, an integrated Red/ET–CRISPR/Cas9 system that enables seamless, marker-free genome editing in marine bacteria. RECC couples Red/ET recombineering with CRISPR/Cas9-mediated cleavage, allowing the incorporation of homology arms and protospacers into a single construct through one-step Gibson assembly, thereby substantially simplifying the engineering process. Using *Pseudoalteromonas flavipulchra* DSM 14401 as a model, we employed RECC to replace the native promoter of a silent nonribosomal peptide synthetase-polyketide synthase (NRPS-PKS) hybrid gene cluster with a strong constitutive promoter. This targeted activation led to the production of a series of previously unknown cyclolipopeptides, designated flavipulchrins. Structural characterization and bioinformatic analysis revealed a plausible biosynthetic pathway for these metabolites. Collectively, RECC provides a robust and generalizable genome-editing platform that facilitates the systematic exploration of biosynthetic potential in genetically recalcitrant marine microorganisms.

## Highlights

• RECC integrates CRISPR/Cas9 with Red/ET for marker-free and electroporation-free gene editing in *Pseudoalteromonas.*
• RECC enables scarless genome editing with an approximately 40 % correct rate, reducing cycle time by 2–3 times.
• Using RECC a cryptic NRPS-PKS BGC in *P. flavipulchra* is activated, revealing two new cyclolipopeptides.

## Graphical abstract

**Figure:**
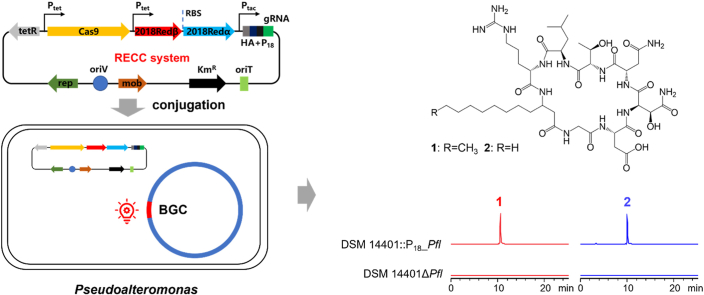

## 1 Introduction

Natural products play a crucial role in drug discovery [1]. Given the serious challenges posed by multidrug resistance, cancer, dementia, etc., humanity urgently needs novel compounds with diverse bioactivities. However, traditional natural product discovery approaches and microbial resources are increasingly proving inadequate due to diminishing returns and high rediscovery rates [2]. Developing a novel approach to exploring microbial resources from understudied environments is an effective strategy [3]. Organisms inhabiting extreme marine environments—such as high pressure, salinity, and low temperature—have evolved unique biosynthetic pathways that produce metabolites with unusual scaffolds. Cheminformatic analyses have revealed that approximately 20–25 % of marine metabolites possess distinct chemical fingerprints not observed in terrestrial analogs, and studies targeting marine specific and understudied microbial phyla result in a higher likelihood of finding marine specific compounds [4]. *Pseudoalteromonas* is an exclusively marine genus widespread in the global ocean, which comprises 20 %–60 % of the marine microbial community. They demonstrate remarkable synthetic potential with the capacity to secrete various enzymes, polysaccharides, and secondary metabolites with industrial biotechnology utility and therapeutic potential [5].

**Fig. 1:**
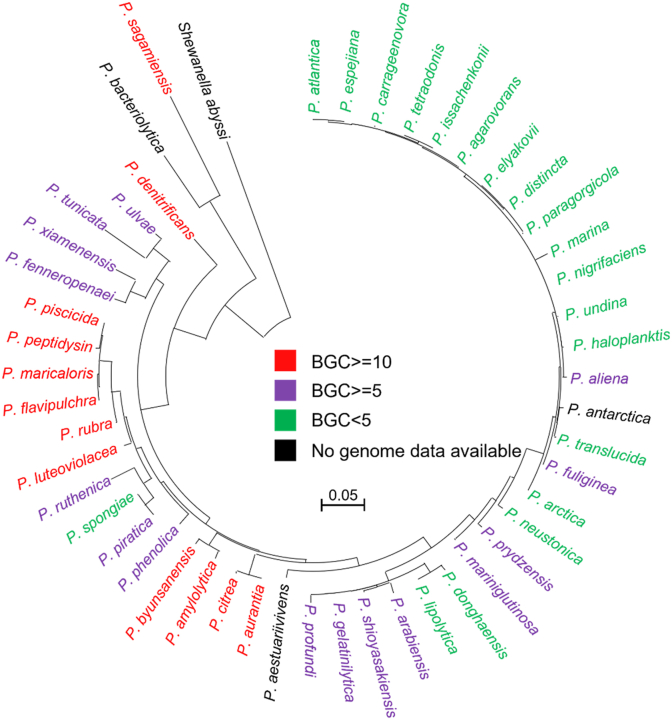
Phylogenetic tree of *Pseudoalteromonas*. The Maximum Likelihood (ML) tree was reconstructed using MEGA 12.0, employing the Tamura-Nei model. The 16S rRNA sequence of the type strain was used to construct the tree and the reference genome was used to evaluate the number of BGC. Red color indicates the number of BGCs≥10, purple color indicates the number of BGCs≥5, green color indicates the number of BGCs<5, and black color indicates the data of the genome is unavailable.

Bioinformatic analysis suggests some *Pseudoalteromonas*, especially the pigmented members, harbor substantial biosynthetic potential (Fig. 1), which devote up to 15 % of their genome to secondary metabolism [6,7]. In the past decade, a few novel compounds with various bioactivities have been isolated and elucidated from *Pseudoalteromonas* through multiple strategies. Ogipeptin A-D were identified in the broth of *Pseudoalteromonas* sp. SANK 71903. They can inhibit lipopolysaccharides (LPS)-induced innate immune reactions through blocking LPS binding to the glycoprotein CD14. Ogipeptins also display activity against *Escherichia coli* [8]. Research on two other strains of *Pseudoalteromonas* resulted in the identification of alterins, a set of CLPs that share the same cationic heptapeptidic cycle with ogipeptins. Alterins can bind to LPS and provoke membrane depolarization and permeabilization, leading to bacterial lysis and potent anti-Gram-negative bacterial activity [9]. Our group also systematically researched natural products from the *Pseudoalteromonas-*based genome mining strategy. Three novel class I lanthipeptides, pseudorosin A-C, originated from *P. flavipulchra* S16, were characterized by heterologous expression [10]. Two silent BGCs were activated through *in situ* promoter replacement, which resulted in the discovery of intramolecular cyclized alteramides and novel ureido-containing linear peptides, respectively [11]. A set of analogs of ulbactin was identified from the fermentation broth of strain S16, and one displayed potent iron-ion chelating activity. Furthermore, through gene knockout and complementation assays, we identified two novel thiazolinyl imine reductases essential for thiazolidine formation [12].

Our previous results suggested that genome mining based on genetic manipulation is an effective way to activate targeted biosynthetic pathways, which could effectively circumvent the problem of compound rediscovery [13]. Although conjugation-based genetic manipulation systems had been established in limited species of *Pseudoalteromonas*, it is still time-consuming and labor-intensive [14,15]. Red/ET-mediated recombineering is a simple and efficient way to perform genetic engineering [16,17]. This system had been successfully applied to a range of bacteria, such as *Salmonella enterica* [18], *Pseudomonas syringae* [19], *Mycobacterium tuberculosis* [20], *Klebsiella pneumoniae* [21], and species of *Photorhabdus* or *Xenorhabdus* [22]. Recently, utilizing Red/ET-mediated recombineering, a series of cryptic BGCs from Burkholderiales species were mined [23,24]. However, the genome editing system based on Red/ET recombinases is electroporation dependent, and the electroporation of marine bacteria presents challenges, which may be due to osmoprotective adaptations, exopolysaccharide matrices, and restriction-modification defense systems evolved in marine environments [15,25].

To overcome this limitation, the Red/ET recombineering was coupled to clustered regularly interspaced short palindromic repeat (CRISPR)/CRISPR-associated protein 9 (Cas9) gene-editing technology. CRISPR/Cas9, which originated from bacterial adaptive immune systems, can introduce highly effective double-strand breaks (DSB) at a target site [26,27]. Given that unrepaired DSB is fatal for cells, CRISPR/Cas9 is a potent counter-selection marker in genetic manipulation. Although the coupling of Red/ET and CRISPR/Cas9 has been reported several times over the past decade, they are all multi-plasmid systems and rely on electroporation for DNA delivery [[28], [29], [30]]. Therefore, these methods are unsuitable for genetic manipulation of *Pseudoalteromonas,* whose electroporation efficiency is low. Here, we developed a single-plasmid approach coupling the Red/ET recombineering with the CRISPR/Cas9 system, which we named RECC system. The newly developed system contains only one plasmid. The Cas9 encoding gene from *Streptococcus pyogenes* and Red/ET recombinases encoding genes from *Pseudoalteromonas* were cloned into one plasmid under the control of an inducible promoter. The guide RNA (gRNA) was driven by the constitutive promoter P_tac,_ and homologous arms were inserted before the spacer sequence; this design allows us to complete the plasmid construction with one step Gibson assembly. Theoretically, once the plasmid was transformed into *Pseudoalteromonas* cells by conjugation, Red/ET recombinases will edit some of the induced cells, and the unedited wild-type cells will be eliminated by Cas9 protein-mediated DSB, which means that the selection marker is unessential in our system.

DSM 14401 is the type strain of *P. flavipulchra* with a 5.44 Mb genome size. The proportion of the genome dedicated to the biosynthesis of secondary metabolites is about 11.8 %, which encodes 15 BGCs containing NRPSs, PKSs, NRPS-PKS hybrid enzymes, and ribosomally synthesized and post-translationally modified peptides (RiPPs) according to antiSMASH [31]. However, most of them are silent under laboratory fermentation conditions. Utilizing the RECC system, one silent NRPS-PKS hybrid BGC of *P. flavipulchra* DSM 14401 was successfully activated. The products of the activated BGC are heptapeptidic cycles with different hydrophobic hydrocarbon tails. The development of RECC enables Red/ET recombineering to edit the genomes of bacteria that are difficult to electroporate, thereby significantly improving the efficiency of gene editing and genome mining. Our study also provides a feasible way to edit the genomes of other difficult-to-electroporate bacterial groups.

## 2 Materials and methods

### 2.1 Bacterial strains and culture conditions

All strains, plasmids, and oligonucleotides used in this study are listed in Supplementary Tables S1–S3. The *E. coli* Turbo cells were cultivated in Luria-Bertani medium (10 g tryptone, 5 g yeast extract, 10 g NaCl) at 37 °C. *E. coli* WM3064 was cultured with LB medium supplemented with 1 mM diaminopimelic acid (DAP). *P. flavipulchra* DSM 14401 was grown in 2216E medium (5 g peptone, 1 g yeast extract, 1 L seawater, pH 7.2) or modified LB medium (MLB, LB medium supplemented with 50 mM MgSO_4_•7H_2_O).

The transformation of *E. coli* Turbo and WM3064 was performed by electroporation [32], while that of DSM 14401 was performed by conjugation [15]. To screen transformants of *E. coli*, kanamycin (Kan) was added to a final concentration of 30 μg/mL. For DSM 14401, the concentration of Kan was 300 μg/mL.

### 2.2 Antibiotic sensitivity assays

Ampicillin (100 μg/mL), kanamycin (50 μg/mL and 300 μg/mL), chloramphenicol (30 μg/mL), erythromycin (10 μg/mL), tetracycline (50 μg/mL), and streptomycin (100 μg/mL) were used for the antibiotic sensitivity assay of DSM 14401. Strain DSM 14401 was grown in MLB broth to the late exponential phase, and then 10 μL of the culture was plated on MLB plates containing each antibiotic. The plates were incubated at 30 °C for up to 3 days, and then the growth condition was observed.

### 2.3 Conjugation procedure

Conjugation experiments were performed as previously described, with some modifications [15]. In brief, donor WM 3064 with plasmid to transform and recipient DSM 14401 were cultured to an OD_600_ value of about 1. Then, 1 mL of culture was collected by centrifugation (9000 rpm for 2 min). The pellets were washed twice with fresh MLB and resuspended in 50 μL MLB. The donor and recipient cells were mixed and dropped on MLB plates with 1 mM DAP. After drying in the clean bench, the plates were incubated at 30 °C for 6 h. One loop of bacterial lawn was picked and spread on MLB plates with 300 μg/mL kanamycin. The plates were incubated at 30 °C for 24 h until clones formed.

### 2.4 Promoter screening

To control the expression of Cas9 and recombinases, four inducible promoters, *P*_*Rha*_, *P*_*tac*_, *P*_*tet*_ and *P*_*BAD*_, were evaluated by Luciferase assays. The general experimental procedure was described in our previous article [11]. To induce the expression of reporter genes, 10 μL anhydrotetracycline (AHT, 20 μg/mL), 1 μL isopropyl β-d-1-thiogalactopyranoside (IPTG, 1 M), 30 μL rhamnose (10 % v/v), or 30 μL arabinose (10 % v/v) was added to 1 mL exponential phase culture of DSM 14401 with different promoters. Then, the light signal was quantified by a microplate reader.

### 2.5 Optimization of electroporation conditions

40 μL of overnight-cultured DSM 14401 was transferred into 1 mL of 2216E, MLB, and LB prepared with 1/3 seawater, respectively. After incubation at 30 °C, 950 rpm for 1 h, 2 h, or 3 h, the cells were collected by centrifugation and washed twice with different electroporation buffers (buffer 1: ddH_2_O, buffer 2: 20 % glycerin, 250 mM sucrose, pH 7.0). The pellets were resuspended in 50 μL of electroporation buffer and electroporated at 1350 V with 1000 ng of plasmid DNA. After electroporation, the cell suspensions were diluted with 1 mL of pre-culture medium and incubated at 30 °C, 950 rpm for 6 h. The cultures were spread on a plate containing 300 μg/mL kanamycin, then the plates were incubated overnight at 30 °C and the number of single clones was counted.

### 2.6 Identification of protein marker

To facilitate the quantification of the efficiency of CRISPR/Cas9 and RECC, a characteristic protein in the supernatant of DSM 14401 culture was selected as a marker. SDS-PAGE was carried out and the marker protein was recovered through gel purification. Then, mass spectrometry was conducted to characterize this marker protein. Briefly, 20 μL of the overnight supernatant of DSM 14401 was mixed with 5 × SDS-PAGE protein loading buffer. The mixture was boiled for 10 min and analyzed using SDS-PAGE. The recovered gel was destained through sonication in 50 μL destain solution (50 % ammonium bicarbonate, 50 % acetonitrile). Then the destain solution was removed, and 50 μL of acetonitrile was added to the gel until the gel turned completely white. Finally, trypsin was used to digest the protein and 50 μL ddH_2_O was used to dissolve the digested products. The sample was analyzed using mass spectrometry after desalting using Ziptip. The encoding gene of the selected marker was identified by searching the Thermo Proteome Discoverer protein database.

### 2.7 Plasmids construction

All plasmids were constructed using Gibson assembly. For the CRISPR/Cas9 editor plasmid, the genes encoding Cas9 and tracRNA were cloned into the backbone of pBBR1-km-oriT under the control of inducible promoter *P*_*tet*_ and constitutive promoter *P*_*tac*_, respectively. A counter-selection marker (*sacB*) was also incorporated to enable plasmid curing, ultimately generating the plasmid pBBR1-km-Ptet-Cas9-SacB-tracRNA. The Red/ET recombinases genes originated from *Pseudoalteromonas* were obtained by BLASTP using a recombineering system from *Pseudomonas aeruginosa* phage Ab31 (BAS) [24]. A recombineering system from *P. agarivorans* Hao 2018 containing Redαβ2018 was synthesized and cloned into plasmid pBBR1-km-P_tet_-Cas9-SacB-tracRNA, generating pBBR1-km-P_tet_-Cas9-Redαβ2018-tracRNA. Two partially complementary oligonucleotides containing the spacer sequence were used as a template and primer for amplification to insert a spacer sequence into the backbone. The gel purification products were assembled with a digested backbone. The homologous arms were obtained and assembled by fusion PCR.

### 2.8 Genome editing analysis of *P. flavipulchra* DSM 14401

To evaluate the editing efficiency, the CRISPR/Cas9 plasmid was transferred into DSM 14401 via conjugation mediated by *E. coli* WM3064. Then, 40 μL of the overnight culture of transformant was transferred to 1 mL MLB medium containing 300 μg/mL kanamycin, and pre-cultured at 30 °C, 950 rpm for 6 h respectively. Subsequently, 10 μL anhydrotetracycline (AHT, 20 μg/mL) was added to induce the expression of Cas9, followed by another 24 h incubation. After that, 50 μL of the culture medium was spread on an MLB agar plate containing kanamycin and incubated at 30 °C overnight. The single clones were picked and cultured for 6 h; the culture medium was then analyzed by SDS-PAGE or colony PCR.

The working conditions of the RECC gene editing system are similar to those of the CRISPR/Cas9 system. Still, the length of the homology arms was optimized and the efficiency of RECC was evaluated by PCR.

### 2.9 Fermentation and analysis of metabolites

The overnight seed culture (1 %, v/v) of *P. flavipulchra* DSM 14401 and its mutants was inoculated to 50 mL of 2216E, CY (casitone 10 g, malt extract 2 g, yeast extract 1 g, seawater 1 L, pH 7.2−7.4), and PYG (peptone 2 g, yeast extract 0.5 g, glucose 4 g, glycerol 5 mL, seawater 1 L, pH 7.2−7.4) broth supplemented with 2 % (v/v) Amberlite XAD-16 resin in 250 mL Erlenmeyer flasks and cultivated at 30 °C, 220 rpm for 24 h. The cells and resin were collected by centrifugation. The pellets were resuspended with 35 mL MeOH and shaken at 200 rpm for 2 h to extract compounds. Then, insoluble materials were removed by centrifugation and MeOH was removed by vacuum evaporation. The residues were dissolved with 1 mL MeOH and 3 μL crude extracts were analyzed by ultrahigh-performance liquid chromatography high-resolution mass spectrometry (UPLC-HRMS) equipped with a C18 column (2.1 × 100 mm, 3.1 μm, Agilent). Analytical program: 0−3 min 5 % solvent B; 3−18 min, 5−95 % solvent B with linear gradient; 18−22 min, 95 % solvent B; 22−22.1 min, 95−5 % solvent B; 22.1−25 min, 5 % solvent B (Solvent A, Milli-Q water supplemented with 0.1 % formic acid; Solvent B, acetonitrile), flow rate: 0.3 mL/min. A Bruker high-resolution Q-TOF mass spectrometer was used to detect compounds in the extracts using positive ionization mode.

### 2.10 Flavipulchrins isolation and purification

To isolate flavipulchrins, large-scale fermentation was carried out with 6 L 2216E broth. The cells and resin were collected by centrifugation. Then, 4 L MeOH was used to elute the compounds adsorbed by the resin, and the resulting crude extract was further separated by Sephadex LH-20 (GE Healthcare) using methanol as the mobile phase. The fractions containing targeted molecular mass were combined, concentrated, and further purified with semipreparative HPLC equipped with a C18 column (Phenomenex Luna, 100 Å, 250 × 10 mm, 10 μm) using the following program: 0−5 min 5 % solvent B; 5−35 min, 5−50 % solvent B with linear gradient; 35−38 min, 50–95 % solvent B; 38−40 min, 95−5 % solvent B; 40−45 min, 5 % solvent B (Solvent A, Milli-Q water supplemented with 0.1 % formic acid; Solvent B, acetonitrile). The ultraviolet (UV) peaks of flavipulchrin A and B were distributed at 30.6 and 28.5 min, respectively (Fig. S1).

### 2.11 Structure elucidation of flavipulchrins

^1^H and ^13^C NMR, DEPT, and 2D NMR spectra of flavipulchrin A and B were recorded in methanol-*d*_*3*_ on a Bruker Avance Neo 600 NMR spectrometer. The structures of flavipulchrin A (**1**) and B (**2**) were elucidated based on NMR and HRMS data, together with comparative analysis of the MS/MS fragmentation patterns of compounds **1** and **2**.

## 3 Results

### 3.1 Establishment of the Red/ET system in *P. flavipulchra* DSM 14401

**Fig. 2:**
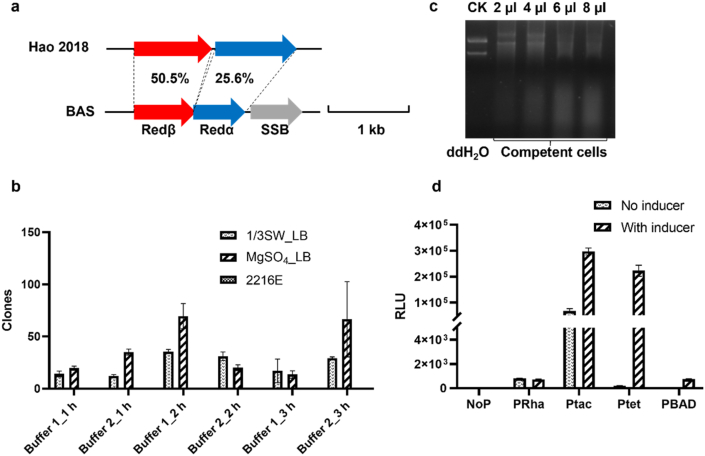
Exploration of conditions for the gene editing system in *Pseudoalteromonas*. (**a**) Recombinase pairs from *Pseudoalteromonas* and its homologs in *Pseudomonas*; (**b**) Electroporation optimization in *P. flavipulchra* DSM 14401; (**c**) The integrity of foreign plasmid DNA after incubation with different volumes of competent cells, the ddH_2_O was used as control (CK); (**d**) Functional performance of distinct inducible promoters in *P. flavipulchra* DSM 14401.

To facilitate the genome mining of *Pseudoalteromonas* with great biosynthetic potential, we initially aimed to establish a convenient gene editing approach using Red/ET recombinases. Given that Red/ET recombinases are typically species-specific [23], we used the previously reported Red/ET system BAS to perform BLAST [24], which led to the identification of three operons containing type Red/ET recombinases across four different *Pseudoalteromonas* strains (Fig. S2). Due to the unavailability of the original strains, the Red/ET system from *P. agarivorans* strain Hao 2018 (Protein ID: AYM89072.1, AYM89073.1) was synthesized for subsequent experiments. Redα2018 (Accession AYM89072.1) is predicted as an endonuclease displaying 25.6 % sequence identity to Redα of the BAS recombineering system, while Redβ2018 (AYM89073.1) is annotated as a phage Bet annealing protein with 50.5 % identity to Redβ (Fig. 2a).

Red/ET recombineering is electroporation-dependent, so we checked the electroporation efficiency of 7 different *Pseudoalteromonas* strains, including *P. rubra* DSM 6842, *P. luteoviolacea* DSM 6061, *P. peptidolytica* DSM 14001, *P. citrea* DSM 8771, *P. piscicida* JCM 20779, *P. flavipulchra* DSM 14401, and *P. flavipulchra* S16. However, most strains we tested are electroporation resistant except *P. flavipulchra* DSM 14401, which showed low susceptibility to electroporation treatment. Subsequently, electroporation conditions (including culture media, buffers, and pre-culture times) were optimized to elevate electroporation efficiency. After incubating in MLB medium at 30 °C for 2 h, and washing twice with electroporation buffer 2, the maximum efficiency was obtained with about 10^2^ CFU/μg DNA (Fig. 2b). The low electroporation efficiency is likely caused by extracellular DNase degradation of plasmid DNA. The plasmid DNA added to a small amount of competent cells (2 μL, 4 μL, 6 μL, and 8 μL) underwent complete degradation within 1 h (Fig. 2c).

To circumvent host toxicity induced by constitutive Red/ET recombinases expression, placing these recombinase genes under an inducible promoter is essential. In this study, we quantitatively assessed induction efficiency and stringency for four common inducible promoters: tetracycline-regulated promoter (*P*_*tet*_) [33], arabinose-regulated promoter (*P*_*BAD*_) [34], isopropyl thiogalactoside-regulated promoter (*P*_*tac*_) [35], and rhamnose-regulated promoter (*P*_*Rha*_) [36], using luciferase assays in DSM 14401. Among these, *P*_*tac*_ demonstrated the highest induction efficiency with relative light unit (RLU) values exceeding 300,000, but its severe leakage (>20 % maximal expression without inducer) precluded practical applications; *P*_*tet*_ exhibited high induction efficiency with RLU values exceeding 200,000 and exceptional stringency (leakage <1 % maximal expression); *P*_*Rha*_ and *P*_*BAD*_ exhibited limited induction efficiency with RLU values below 1000 (Fig. 2d). Therefore, *P*_*tet*_ was utilized to control Redαβ2018 expression in DSM 14401.

Antibiotic sensitivity assays were performed to identify suitable selection markers for screening DSM 14401 transformants. No obvious colony was observed on MLB plates with 300 μg/mL kanamycin, 30 μg/mL chloramphenicol, or 10 μg/mL erythromycin after 3 days of incubation (Table S4). Therefore, these three resistance genes are promising candidates for selection markers for transformant screening and gene knockout in DSM 14401.

The synthesized genes of Redαβ2018 were cloned into the backbone of pBBR1-km under the control of *P*_*tet*_, generating the plasmid pBBR1-km-P_tet_-Redαβ2018. The resulting plasmid was introduced into DSM 14401 cells by electroporation, which resulted in DSM 14401/Redαβ2018. We then tested the recombination efficiency between linear DNA and the genomic DNA of DSM 14401. 120 bp homologous arms targeted to non-essential BGC were flanked to the erythromycin marker using synthesized oligonucleotides and PCR amplification. Then, the constructed fragment was introduced into DSM 14401/Redαβ2018 cells by electroporation. However, no recombinant was obtained, which may be due to the low electroporation efficiency. The typical frequency of recombinants is one positive clone out of 10^4^ to 10^5^ colonies. Despite optimization, the maximum efficiency remained below 10^2^ CFU/μg DNA (Fig. 2b), which failed to reach the threshold essential for effective Red/ET recombineering.

### 3.2 Establishment and optimization of the RECC system in *P. flavipulchra* DSM 14401

**Fig. 3:**
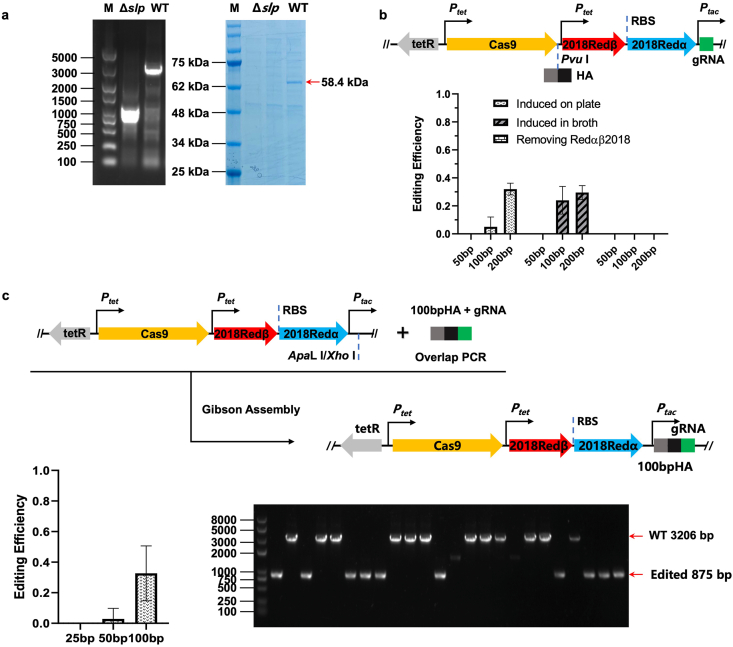
Establishment of the RECC gene editing system in *P. flavipulchra* DSM 14401. (**a**) The marker protein expressed by the *slp* gene; (**b**) The editing efficiency of the RECC system with homologous arms located between Cas9 and Redαβ2018; (**c**) One-step Gibson assembly of RECC plasmid with homologous arms adjacent to the 5′ end of sgRNA and its editing efficiency.

To circumvent the reliance of Red/ET recombineering on electroporation, the CRISPR/Cas9 system was coupled with Redαβ2018 for counterselection to eliminate unmodified cells. We first evaluated the editing efficiency of CRISPR/Cas9 in DSM 14401 cells. To facilitate a more straightforward and visually quantifiable assessment of CRISPR/Cas9 editing efficiency, a highly abundant protein in the DSM 14401 supernatant was selected as a marker. Using protein mass spectrometry analysis, this marker was identified as a surface-layer protein (S-layer protein, SLP). This identification was further verified through gene knockout experiments. The gene encoding SLP was knocked out using a previously described method [12], resulting in the marker's disappearance from the supernatant of the DSM 14401ΔSLP strain (Fig. 3a).

The spacer sequence targeted gene encoding SLP was cloned into the resulting plasmid, generating CRISPR editor plasmid pBBR1-km-P_tet_-Cas9-SacB-SLP (Fig. S3). The CRISPR plasmid was transformed into DSM 14401 through conjugation. After pre-incubation and induction, most strains we tested were successfully edited and the SLP protein disappeared in their supernatants (Fig. S3), which suggested that CRISPR/Cas9 can perform highly efficient counter-selection in DSM 14401. However, we also observed unstable editing efficiency across independent biological replicates, potentially resulting from CRISPR escape. While the exact mechanisms underlying CRISPR escape remain incompletely understood, several potential mechanisms have been proposed, such as the mutation in target DNA regions [37,38], the destruction of Cas protein [39,40] and the deletion of the spacer region on the sgRNA resulting from the recombination of repeat sequences [28]. To explore possible mechanisms underlying CRISPR escape, we firstly assessed Cas9 gene integrity in CRISPR escapers via PCR; however, no significant deletion or insertion within the Cas9 coding sequence was detected among selected strains (Fig. S4); we also sequenced the protospacer regions of CRISPR escapers and no mutation was detected (Fig. S5). Based on these results, we speculated that DSM 14401 escapes the CRISPR/Cas9 system through an unknown pathway. Introducing the Red/ET system may provide an easier way for CRISPR escape, which means that the coupling of Red/ET and CRISPR/Cas may improve editing efficiency.

Subsequently, plasmids pBBR1-km-P_tet_-Cas9-Redαβ2018-SLP with homologous arms in different lengths were constructed and transformed into DSM 14401 to knockout the SLP gene (Fig. 3b). After pre-incubation and induction in MLB broth, the editing efficiency was detected by PCR. No edited colonies were obtained with homologous arms ≤50 bp, whereas editing efficiency exceeded 20 % when arm length increased to 100 bp. Further extension of the length of homologous arms resulted in a slight increase in editing efficiency (Fig. 3b). We also performed induction directly on MLB plates supplemented with inducer AHT. Although the editing efficiency using 200 bp homologous arms reached ∼30 %, similar to the efficiency achieved by induction in MLB broth (Fig. 3b), the colony yield was significantly reduced. In some experimental replicates, even no colonies were obtained. Therefore, the system was induced in broth in the following experiments. We also constructed a plasmid with the Redαβ2018 system removed while retaining the 50, 100, or 200 bp homologous arms. No edited colonies were obtained using the resulting plasmids, demonstrating that the Redαβ2018 system is essential for recombination (Fig. 3b).

Initially, the homologous arms were cloned between the Cas9 gene and Redβ2018 (Fig. 3b), meaning the editor plasmid needs to be constructed in two rounds of Gibson assembly. To facilitate plasmid construction, the homologous arms were cloned upstream of the spacer sequence. The DNA fragments containing spacer sequences and homologous arms were constructed by fusion PCR and cloned into the digested backbone in one round of assembly. To test whether 5′ extensions impair CRISPR/Cas9 functionality, we fused a 200 bp sequence upstream of the SLP spacer and constructed plasmid pBBR1-km-P_tet_-Cas9-SacB-200bp-SLP. This construct maintained high editing efficiency in DSM 14401 cells (Fig. S6), demonstrating tolerance for extensive 5′ terminal spacer fusions without functional compromise. Then, homologous arms and the SLP spacer were cloned to plasmid pBBR1-km-P_tet_-Cas9-Redαβ2018 in one round of Gibson assembly. Moreover, the editing efficiency was detected by colony PCR. Consistent with previous observations, editing efficiency exceeded 20 % when using 100 bp homologous arms. We isolated limited edited colonies with 50 bp arms, whereas no edited colonies were obtained using 25 bp homologous arms (Fig. 3c). This system, coupled with Red/ET recombineering and CRISPR/Cas9, was named RECC.

### 3.3 RECC-mediated activation of cryptic BGC in *P. flavipulchra* DSM 14401

**Fig. 4:**
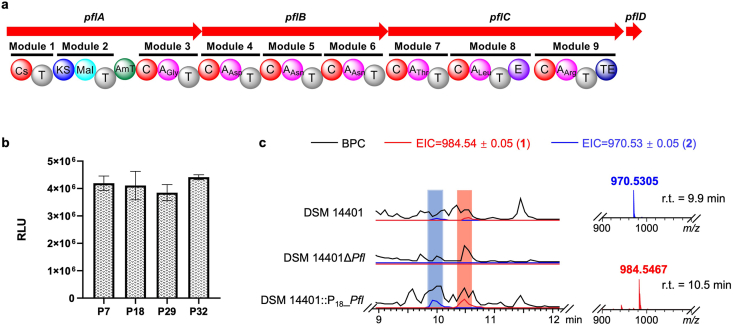
Utilizing the RECC system to activate cryptic biosynthetic gene clusters in *P. flavipulchra* DSM 14401. (**a**) The composition of biosynthetic gene cluster 1–4; (**b**) Functional performance of distinct promoters in *P. flavipulchra* DSM 14401; (**c**) LC-MS analysis of wild type *P. flavipulchra* DSM 14401 and its BGC1-4 inactivation and activation mutants. BPC: Base Peak Chromatogram, EIC: Extracted Ion Chromatogram.

Following the successful establishment of an efficient and straightforward RECC gene editing system, we implemented promoter optimization strategies to activate cryptic BGCs in DSM 14401. DSM 14401 contains abundant uncharacterized secondary metabolite BGCs, including thiopeptide, lanthipeptide, NRPS, NRPS-PKS hybrid and other types of BGCs (Table S5). BGC1-4, which we named the *Pfl* cluster, is a hybrid NRPS-PKS cluster predicted to encode lipopeptide biosynthesis. antiSMASH analysis indicates a putative heptapeptide core structure containing: glycine, aspartic acid, two asparagines, threonine, non-proteinogenic d-leucine, and phenylalanine (Fig. 4a). Lipopeptides biosynthesized by NRPS constitute a structurally diverse class of natural products with broad and potent biological activities. Many of these molecules exhibit strong antimicrobial properties, including the clinically used antibiotics daptomycin and polymyxins. Therefore, investigating the *Pfl* gene cluster not only expands the structural diversity of lipopeptides, but also holds the potential to uncover new antibiotic candidates.

Subsequently, the *Pfl* cluster was activated by promoter replacement using the RECC system developed in this study. The promoter efficiency in DSM 14401 of four potent constitutive promoters (*P*_*7*_, *P*_*18*_, *P*_*29*_, *P*_*32*_) from *P. flavipulchra* S16 reported in our previous study [11] was evaluated using luciferase assays (Fig. 4b). Since all four promoters proved efficient, *P*_*18*_ was ultimately chosen to activate the *Pfl* cluster. The recombinants with the replacement of the original promoter of *Pfl* by potent promoter *P*_*18*_ (designated DSM 14401::P_18__*Pfl*) were obtained with a successful rate of >50 %. Meanwhile, *Pfl* cluster inactivated strains (DSM 14401Δ*Pfl*) were successfully obtained via CRISPR/Cas9-mediated non-homologous end joining (NHEJ). However, DSM 14401Δ*Pfl* mutants generated via single-guide RNA (sgRNA)/Cas9 in this study are a fragment deletion of the targeted sequence rather than base insertion-deletion (indel), which is observed in eukaryotic cells (Fig. S7) [41,42]. Sanger sequencing of the PCR products revealed that all three DSM 14401Δ*Pfl* mutants shared an identical genotype, characterized by a 10.5-kb deletion encompassing the protospacer region. (Fig. S8).

Then, wild-type DSM 14401, the *Pfl* cluster inactivation mutant (DSM 14401Δ*Pfl*) and the *P*_*18*_ promoter replacement strain (DSM 14401::P_18__*Pfl*) were fermented in parallel using 2216E broth, PYG broth and CY broth. Crude extracts isolated by adsorption on XAD-16 resin were subjected to UPLC-HRMS analysis. Compared to DSM 14401Δ*Pfl*, a series of peaks with distinct *m/z* values was detected in the crude extract of DSM 14401::P_18__*Pfl* (Fig. 4c and S9). Detailed comparative analysis of LC-MS chromatograms revealed that these differential peaks are present, albeit at lower intensities, in wild-type DSM 14401 crude extracts (Fig. 4c and S9). This indicates basal-level expression of the *Pfl* gene cluster under standard laboratory fermentation conditions.

### 3.4 Structure elucidation of lipopeptide flavipulchrins

Targeted isolation and purification of high-abundance constituents from the *Pfl* gene cluster metabolites was subsequently performed. The 6 L fermentation broth crude extract was fractionated on a Sephadex LH-20 column using methanol as eluent. Fractions containing target compounds were pooled and further purified by semipreparative HPLC. Approximately 20 mg of compound **1** was obtained as a white powder, and the molecular formula was established as C_42_H_74_N_13_O_14_, based on HR-ESIMS (*m/z* 984.5467 [M + H]^+^, calcd 984.5473). Initial structural characterization of **1** via NMR spectroscopy in DMSO‑*d*_6_ revealed poorly resolved signals in ^1^H and ^13^C spectra (Fig. S10), likely attributed to intramolecular hydrogen bonding or prototropic exchange. Due to the limited solubility of **1** (only in DMSO and methanol), and considering that fully deuterated methanol-*d*_*4*_ (CD_3_OD) would cause solvent-solute proton exchange leading to the disappearance of exchangeable proton signals, we selected methanol-*d*_*3*_ (CD_3_OH) as the solvent for NMR spectroscopic analysis, which significantly improved spectral resolution (Fig. S11).

**Fig. 5:**
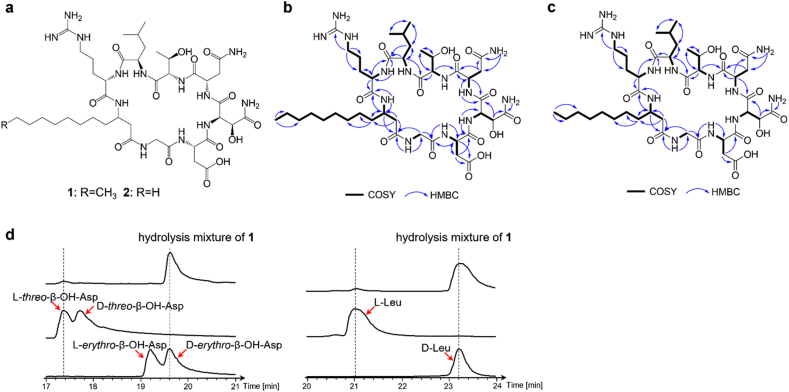
Structure elucidation of flavipulchrin A (**1**) and B (**2**). (**a**) The structure of flavipulchrin A (**1**) and B (**2**); (**b**) Key COSY and HMBC correlations of flavipulchrin A (**1**); (**c**) Key COSY and HMBC correlations of flavipulchrin B (**2**); (**d**) LC-MS analysis of the stereo configuration of β–OH–Asp (EIC = 402.1) and Leu (EIC = 384.1).

The ^1^H, ^13^C, DEPT, and HSQC NMR spectra of **1** (Table S6, Fig. S11) showed signals for 42 distinct carbon signals, including 11 carbonyl carbons (*δ*_C_ 178.5, 175.7, 175.1, 175.0, 174.8, 174.5, 174.3, 174.0, 173.3, 171.8, 170.8), one guanidyl carbon (*δ*_C_ 158.7), 10 methines with two oxygenated signals (*δ*_H/C_ 4.45/72.4 and 4.27/69.8) and 7 nitrogenated carbons (*δ*_H/C_ 4.43/60.3, 4.56/59.5, 4.23/54.8, 4.60/54.1, 4.45/53.8, 4.76/52.1, 4.15/48.9), 16 methylenes (two nitrogenated: *δ*_H/C_ 3.95, 3.68/44.2, 3.15/42.2), 4 methyls (*δ*_H/C_ 0.95/23.9, 0.85/21.0, 1.19/19.8, 0.89/14.3). The 2D NMR data (Fig. 5b) combined with the substrate specificity predicted by the adenylation (A) domain (Fig. 4a) revealed the existence of six standard amino acids: Gly, Asp, Asn, Thr, Leu, and Arg. The OH-Asn was established via the COSY correlations of OH-Asn H-2 (*δ*_H_ 4.56) and H-3 (*δ*_H_ 4.45) together with HMBC correlations from H-3 to C-1 (*δ*_C_ 170.8) and C-4 (*δ*_C_ 175.7). Similarly, the 3-aminododecanoic acid (3-Ada) unit was also confirmed by the successive COSY correlations between 3-Ada H-2 (*δ*_H_ 2.45, 2.22) to H-12 (*δ*_H_ 0.89) and HMBC cross-peaks from H-2/H-3 (*δ*_H_ 4.15) to C-1 (*δ*_C_ 175.0). Finally, these eight residues were connected to form a cyclic polypeptide by detailed interpretation of the HMBC correlations from the amide and its adjacent protons to neighboring carbonyl groups (Fig. 5b).

The molecular formula of compound **2** was established as C_41_H_72_N_13_O_14_, based on HR-ESIMS (*m/z* 970.5305 [M + H]^+^, calcd 970.5316). The NMR spectra of compound **2** (Table S7, Fig. S12) are highly similar to those of compound **1**, except that **2** lacks a methylene signal. The 2D NMR data (Fig. 5c) revealed that the 3-Ada tail in **1** is replaced by a shorter 3-aminoundecanoic acid (3-Aua) in **2**, which was also supported by MS/MS analysis (Fig. S13). The MS/MS signal at *m/z* 156.1747 (observed) was assigned to the characteristic fragments [CH_3_(CH_2_)_8_CH=NH_2_]^+^ (calcd 156.1747) for compound **1**. In contrast, compound **2** exhibited the corresponding fragment at *m/z* 142.1588 (observed), consistent with [CH_3_(CH_2_)_7_CH=NH_2_]^+^ (calcd 142.1590). We also observed other peptidyl fragments in the MS/MS spectra of **1** and **2**, such as [Gly^1^-Asp^2^-(β–OH–Asn)^3^-Asn^4^-Thr^5^-Leu^6^-Arg^7^]^+^ at *m/z* 787.3705 (calcd 787.3693), [Asp^2^-(β–OH–Asn)^3^-Asn^4^-Thr^5^-Leu^6^-Arg^7^]^+^ at *m/z* 730.3491 (calcd 730.3478), [(β–OH–Asn)^3^-Asn^4^-Thr^5^-Leu^6^-Arg^7^]^+^ at *m/z* 615.3205 (calcd 615.3209). The fragments with 3-aminofatty acyl were also detected in MS/MS spectra of **1** and **2**, such as [Leu^6^-Arg^7^-3-aminofatty acid- Gly^1^-Asp^2^-(β–OH–Asn)^3^] at *m/z* 769.4584 of **1** versus 755.4403 of **2**, [Asn^4^-Thr^5^-Leu^6^-Arg^7^-3-aminofatty acid- Gly^1^] at *m/z* 739.4845 of **1** versus 725.4666 of **2** (Fig. S13). The MS/MS data, together with the NMR data, further confirmed the structures of compounds **1** and **2**.

The absolute configurations of amino acid residues of **1** were identified by Marfey's assay combined with the presence of E domains and the type of following C domains. Based on the results of Marfey's assays (Fig. 5d and S14), the Asp, Asn, Thr and Arg residues are in the L-configuration, and the Leu residue is in the D configuration. To determine the absolute configuration of β–OH–Asn in compound **1**, commercially available *cis*- and *trans*-epoxysuccinic acid (MACKLIN™) were subjected to ammonolysis, yielding hydroxyaspartic acid diastereomers for stereochemical analysis. LC-MS analysis revealed two β-hydroxyasparagine diastereomers in the acid hydrolysate: (*2S*,*3S*) *threo*-β-hydroxy-l-asparagine and (*2R*,*3S*) *erythro*-β-hydroxy-d-asparagine (Fig. 5d). A similar phenomenon was also observed when determining the leucine configuration (Fig. 5d), which may result from configuration inversion at Cα during acid hydrolysis. To our surprise, the peak corresponding to d-*erythro*-β–OH–Asp accounted for a larger proportion, indicating that the stereochemical configuration of β–OH–Asp is (*2R*,*3S*). However, module 5 lacks an epimerization (E) domain. To elucidate the biosynthesis of d-β–OH–Asn, we further determined the subtype of the C domain through phylogenetic analysis [43]. Both the C domains in module 5 (C5) and module 6 (C6) belong to the ^L^C_L_ subtype rather than the dual E/C subtype or ^D^C_L_ subtype (Fig. S15), which makes the biosynthesis of d-β–OH–Asn more confusing. The biosynthesis of d-β–OH–Asn within the *Pfl* gene cluster appears to represent a previously uncharacterized pathway, the elucidation of which warrants further investigation. Based on the results of NMR, MS/MS, and Marfey's assay, the stereochemical structures of compounds **1** and **2** were established as shown in Fig. 5a.

### 3.5 Proposed biosynthetic pathway of flavipulchrins

**Fig. 6:**
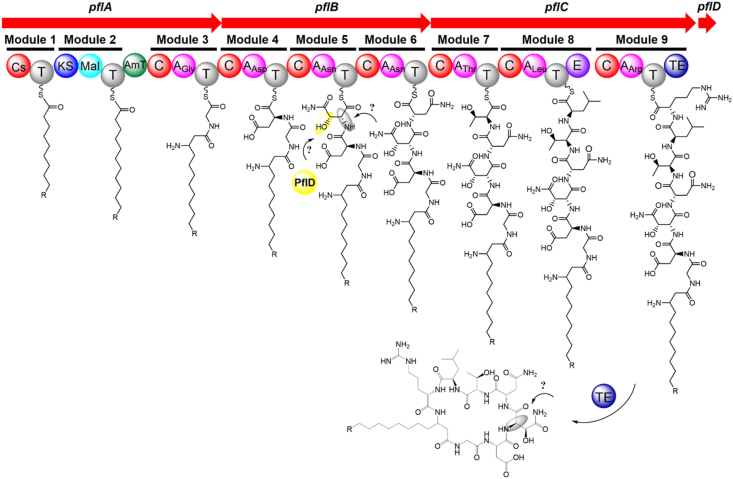
Proposed biosynthetic pathway of flavipulchrins.

Combining the BGC and the structures of **1** and **2**, we proposed the biosynthetic pathway of flavipulchrins. The process begins with the Cs domain introducing nonanoic acid or decanoic acid as a starter unit for flavipulchrin biosynthesis. The PKS module then elongates the chain by introducing a C_2_ unit derived from malonyl-CoA. Subsequent installation of an amino group at C-3 is mediated by an aminotransferase domain. NRPS modules sequentially incorporate glycine, aspartate, two asparagines, threonine, leucine, and arginine. Among them, PflD, a free-standing TauD/TfdA family dioxygenase, may be involved in β-carbon hydroxylation of Asn-3. Phylogenetic analysis demonstrated that PflD is closely related to the dioxygenases involved in the biosynthetic pathways of clovibactin and hypeptin (Fig. S16) [44,45]. HynE can hydroxylate the Asn residue attached to the T domain, resulting in the formation of β–OH–Asn in hypeptin [45], which indicates that the hydroxylation of Asn-3 in flavipulchrins likely occurs online. However, the mechanism underlying the epimerization of Asn-3 remains mysterious. In contrast, Leu-6 undergoes epimerization to the D-configuration by an epimerase (E) domain in module 8. Finally, the thioesterase (TE) domain catalyzes macrolactam formation through cyclization between the C-terminus of Arg-7 and the C-3 amino group, yielding compounds **1** and **2** (Fig. 6).

### 3.6 Biological activity assay

The antimicrobial activity of compound **1** and **2** was evaluated using the disc diffusion method. However, it exhibited no activity against selected strains, including *Pseudomonas aeruginosa* PAO1, *E. coli* GB05, *Bacillus subtilis* 0047, two *Vibrio* strains (*V. alginolyticus* ATCC 17749 and *Vibrio* sp. J10), and two *Pseudoalteromonas* strains (*P. agarivorans* DSM 14585 and *P. issachenkonii* KMM 3549), even at a concentration up to 1 mM.

## 4 Discussion

As an exclusively marine genus, the members of *Pseudoalteromonas* have evolved unique biosynthetic pathways to adapt to marine environments. However, due to the lack of gene editing tools, the genome mining of this group remains underexplored. An effective and convenient gene editing tool is crucial for genome mining of this understudied non-model bacterial group. However, current research on gene editing primarily focuses on conventional model bacteria, such as *Escherichia coli*, *Streptomyces*, and *Bacillus*, while studies on non-model bacteria, particularly those from marine environments, remain limited.

In this study, we successfully performed scarless gene editing in *Pseudoalteromonas* utilizing the newly developed electroporation-independent gene editing system. This innovative system streamlines the workflow considerably compared to existing editing tools, significantly reducing experimental time and labor requirements, enabling targeted gene deletions and insertions within a week. By employing this system, we replaced the native promoter of the biosynthetic gene cluster BGC1-4 in *P. flavipulchra* DSM 14401 with a robust promoter derived from *P. flavipulchra* S16, leading to the identification of two novel cyclolipopeptides, named flavipulchrin A and B. Given the extensive biosynthetic capabilities of *Pseudoalteromonas*, this advanced gene editing technology dramatically enhances the genome mining efforts within this genus. Although several natural products from *Pseudoalteromonas* have been documented in recent literature, these findings merely scratch the surface of its vast biosynthetic potential.

Since *Pseudoalteromonas* predominantly thrives in marine environments, the unique selective pressures of such ecosystems have likely influenced the evolution of specialized enzymatic processes and metabolic products. In the present study, we have identified a novel pathway responsible for the biosynthesis of a d-amino acid in *Pseudoalteromonas*. Based on current reports, we hypothesize that the epimerization and hydroxylation of Asn-3 are both mediated by PflD, potentially through a desaturation step followed by hydrolytic transformation. As a member of the Fe(II)/α-ketoglutarate-dependent oxygenase family, its homologous enzymes display a broad range of catalytic activities, including hydroxylation, desaturation, epoxidation, skeletal rearrangement, and epimerization, reflecting their remarkable functional diversity [46,47]. While this hypothesis and the catalytic mechanism of PflD remain to be elucidated, we cannot exclude the possibility that Asn-3 epimerization may be mediated by other pathways. Nevertheless, our findings indicate that marine *Pseudoalteromonas* possess numerous unexplored and novel biosynthetic pathways that merit further examination. We anticipate that ongoing genome mining efforts utilizing the RECC system will reveal diverse natural products exhibiting distinctive structural modifications, thereby presenting significant biotechnological opportunities.

We also expect to apply this newly developed RECC system to other non-model microorganisms that currently lack efficient gene-editing tools, thereby unlocking more of the hidden secrets within the microbial world. Like *Pseudoalteromonas*, countless non-model bacteria—thriving in distinct environments, engaging in unique symbiotic relationships, or possessing unparalleled metabolic capabilities—remain genetically intractable. Their study has been hampered by the absence of robust, species-specific genetic manipulation systems. Our technology, which is independent of electroporation and selection markers, has the potential to bridge this critical gap. It will empower researchers to systematically dissect novel metabolic pathways for the sustainable production of biofuels, pharmaceuticals, and biomaterials directly from these untapped biological resources.

To broaden its applicability, further optimization to enhance the efficiency of the RECC system is still required. The unedited colonies obtained using the RECC system may be attributable to CRISPR escape mutants in *P. flavipulchra* DSM 14401. Initial characterization of CRISPR evasion strategies in *P. flavipulchra* DSM 14401 leveraged previously documented escape paradigms. However, the observed CRISPR escape in DSM 14401 is inconsistent with established mechanisms [37,38], indicating a previously unidentified evasion strategy. Further characterization of the novel CRISPR evasion mechanisms in DSM 14401, or the identification of a more compatible CRISPR/Cas system, could enhance the editing efficiency of the RECC system. Moreover, discovering more efficient Red/ET recombinases or fine-tuning the expression of the Red/ET and CRISPR/Cas systems may further improve its overall efficiency.

This study established a novel RECC gene-editing platform by coupling the Red/ET recombination system with CRISPR/Cas9. This approach eliminates the need for electroporation and selection markers while enabling scarless genome editing. Functional validation of RECC in *Pseudoalteromonas* demonstrates its potential to accelerate genome mining in this underexplored lineage with substantial biosynthetic capacity. Specifically, RECC-mediated promoter engineering in *P. flavipulchra* DSM 14401 successfully activated a cryptic biosynthetic gene cluster, discovering structurally novel metabolites. These results position RECC as an efficient framework for targeted genome mining in genetically intractable *Pseudoalteromonas* strains. Looking forward, more profound insights into escape mechanisms will advance our understanding of host–CRISPR/Cas interactions, informing the design of more robust gene-editing tools. Beyond activating silent gene clusters, RECC opens new avenues for fundamental and applied research in genetically intractable bacteria.

## CRediT authorship contribution statement

**Zong-jie Wang:** Writing – review & editing, Writing – original draft, Validation, Investigation, Formal analysis, Data curation, Conceptualization. **Haibo Zhou:** Validation, Software, Data curation. **Youming Zhang:** Supervision. **Fu Yan:** Supervision. **Liujie Huo:** Writing – review & editing, Writing – original draft, Validation, Supervision, Conceptualization. **Xiaotong Wang:** Writing – review & editing, Writing – original draft, Validation, Methodology, Investigation.

## Declaration of competing interest

The authors declare that they have no known competing financial interests or personal relationships that could have appeared to influence the work reported in this paper.
