# Supplementary material for: RECC: A Red/ET–CRISPR/Cas9-based system enabling genome mining of marine Pseudoalteromonas for novel natural products
Source: Synth Syst Biotechnol. 2026 Jan 10;12:342–51. doi: 10.1016/j.synbio.2025.12.015 (PMC12819058; doi:10.1016/j.synbio.2025.12.015)
Supplement: Multimedia component 1 [file mmc1.docx]

**RECC: A Red/ET–CRISPR/Cas9-Based System Enabling Genome Mining of Marine *Pseudoalteromonas* for Novel Natural Products**

Zong-jie Wang^1^, Haibo Zhou^1^, Youming Zhang^1^, Fu Yan^2*^, Liujie Huo^1*^, Xiaotong Wang^1*^

1. Helmholtz International Laboratory, State Key Laboratory of Microbial Technology, Shandong University, Qingdao 266237, P. R. China.
2. Hunan Provincial Key Laboratory of Microbial Molecular Biology, College of Life Science, Hunan Normal University, Changsha 410081, China.

*Corresponding emails: wangxt@sdu.edu.cn, lhuo@sdu.edu.cn, fuyan@hunnu.edu.cn

**Table S1.** List of strains used in this study.

| **Strain** | **Description** | **Ref.** |
| --- | --- | --- |
| *E. coli* Turbo | *E. coli* cells suitable for high efficiency transformation and rapid colony growth | NEB |
| *E. coli* WM3064 | *E. coli* donor strain used for conjugation | ^1^ |
| *P. flavipulchra* DSM 14401 | The type strain of *P. flavipulchra* | Gifted by Prof. Su from sdu |
| DSM 14401Δ*slp* | Deletion of the surface-layer protein gene (*slp*) in DSM 14401 | This study |
| DSM 14401Δ*Pfl* | Deletion of the BGC of flavipulchrin in DSM 1440 | This study |
| DSM 14401::P_18__*Pfl* | Replacement of the native promoter of the flavipulchrin BGC in DSM 14401 with the strong constitutive promoter P_18_ | This study |

**Table S2.** List of plasmids used in this study.

| **Plasmid** | **Description** | **Ref.** |
| --- | --- | --- |
| pBBR1-km^R^-oriT-*Eco*RI-firefly | Plasmid used for luciferase assays in DSM 14401 | This study |
| pBBR1-km^R^-oriT-P_tac_-firefly | Reporter gene regulated by promoter P_tac_ | This study |
| pBBR1-km^R^-oriT-P_tet_-firefly | Reporter gene regulated by promoter P_tet_ | This study |
| pBBR1-km^R^-oriT-P_BAD_-firefly | Reporter gene regulated by promoter P_BAD_ | This study |
| pBBR1-km^R^-oriT-P_Rha_-firefly | Reporter gene regulated by promoter P_Rha_ | This study |
| pBBR1-km^R^-oriT-P_7_-firefly | Reporter gene promoted by potent promoter P_7_ | This study |
| pBBR1-km^R^-oriT-P_18_-firefly | Reporter gene promoted by potent promoter P_18_ | This study |
| pBBR1-km^R^-oriT-P_29_-firefly | Reporter gene promoted by potent promoter P_29_ | This study |
| pBBR1-km^R^-oriT-P_32_-firefly | Reporter gene promoted by potent promoter P_32_ | This study |
| pBBR1-km^R^-oriT-P_tet_-Redαβ2018 | Recombinases Redαβ2018 regulated by promoter P_tet_ | This study |
| pBBR1-km^R^-oriT-P_tet_-Cas9 | CRISPR/Cas9 system regulated by promoter P_tet_ | This study |
| pBBR1-km^R^-oriT-P_tet_-Cas9-*slp* | CRISPR/Cas9 system targeted *slp* gene | This study |
| pBBR1-km^R^-oriT-P_tet_-Redαβ2018-Cas9 | CRISPR/Cas9 system coupling with Recombinases Redαβ2018 (RECC System) | This study |
| pBBR1-km^R^-oriT-P_tet_-Redαβ2018-Cas9-*slp*-*Pvu*I-50bp | RECC system targeted *slp* gene with 50 bp homologous arms (HAs) located between Cas9 and Redαβ2018 | This study |
| pBBR1-km^R^-oriT-P_tet_-Redαβ2018-Cas9-*slp*-*Pvu*I-100bp | RECC system targeted *slp* gene with 100 bp HAs located between Cas9 and Redαβ2018 | This study |
| pBBR1-km^R^-oriT-P_tet_-Redαβ2018-Cas9-*slp*-*Pvu*I-200bp | RECC system targeted *slp* gene with 200 bp HAs located between Cas9 and Redαβ2018 | This study |
| pBBR1-km^R^-oriT-P_tet_-Cas9-100bp-*slp* | CRISPR/Cas9 system targeted *slp* gene with 200 bp bases adjacent to the 5’ end of sgRNA | This study |
| pBBR1-km^R^-oriT-P_tet_-Redαβ2018-Cas9-25bp-*slp* | RECC system targeted *slp* gene with 25 bp HAs located before sgRNA | This study |
| pBBR1-km^R^-oriT-P_tet_-Redαβ2018-Cas9-50bp-*slp* | RECC system targeted *slp* gene with 50 bp HAs located before sgRNA | This study |
| pBBR1-km^R^-oriT-P_tet_-Redαβ2018-Cas9-100bp-*slp* | RECC system targeted *slp* gene with 100 bp HAs located before sgRNA | This study |
| pBBR1-km^R^-oriT-P_tet_-Cas9-BGC1-4 | CRISPR/Cas9 system used for destroying BGC 1-4 | This study |
| pBBR1-km^R^-oriT-P_tet_-Redαβ2018-Cas9-P_18_-BGC1-4 | RECC system used for replacing the native promoter of BGC 1-4 with P_18_ | This study |

**Table S3.** List of primers used in this study.

| **Primer** | **Primer sequence (5'-3')** | **Application** |
| --- | --- | --- |
| BBR1-F | GGATCCTCTAGACCCAGCCC | Used for constructing plasmid pBBR1-kmR-oriT-*Eco*RI-firefly |
| BBR1-kmR-R | TGCATAAAAACTGTTGTAATTCAT |  |
| HA-oriT-F | ATGAATTACAACAGTTTTTATGCAGTGCACCGGCCAGCCTCGCAGAGCA |  |
| HA-*EcoR*I-oriT-R | CTTTATGTTTTTGGCGTCTTCCAT*GAATTC*GGGCAGGATAGGTGAAGTAG |  |
| Firefly-F | ATGGAAGACGCCAAAAACATAAAG |  |
| HA-Firefly-R | CTCATTAGGCGGGCTGGGTCTAGAGGATCCTTACAATTTGGACTTTCCGCCC |  |
| HA-P_tac_-firefly-F | CTACTTCACCTATCCTGCCCGTGAAACCAGTAACGTTATAC | Used for constructing plasmid pBBR1-kmR-oriT-firefly with different promoters |
| HA-P_tac_-firefly-R | TCTTTATGTTTTTGGCGTCTTCCATCATATGAATTCCTCCTGTGTGA |  |
| HA-P_tet-_firefly-F | CTACTTCACCTATCCTGCCCTTAAGACCCACTTTCACATTTAAG |  |
| HA-P_tet_-firefly-R | TCTTTATGTTTTTGGCGTCTTCCATATGAATTCTCTCTATCACTGATAG |  |
| HA-P_BAD_-firefly-F | CTACTTCACCTATCCTGCCCTTATGACAACTTGACGGCTAC |  |
| HA-P_BAD_-firefly-R | TCTTTATGTTTTTGGCGTCTTCCATAGTGAATTCCTCCTGCTAGC |  |
| HA-P_Rha_-firefly-F | CTACTTCACCTATCCTGCCCTTAATCTTTCTGCGAATTGAG |  |
| HA-P_Rha_-firefly-R | TCTTTATGTTTTTGGCGTCTTCCATATGTATATCTCCTTCTTAAGAATTG |  |
| HA-P_7_-firefly-F | CTACTTCACCTATCCTGCCCAAAGTAACAACTAAATAATTGTC |  |
| HA-P_7_-firefly-R | TCTTTATGTTTTTGGCGTCTTCCATTTTTATCTCCATGTATCCGG |  |
| HA-P_18_-firefly-F | CTACTTCACCTATCCTGCCCATATCAATCTTTAAATAACTATTGG |  |
| HA-P_18_-firefly-R | TCTTTATGTTTTTGGCGTCTTCCATTTGTGTTCCCTAATCTAGGT |  |
| HA-P_29_-firefly-F | CTACTTCACCTATCCTGCCCAATAAATATAGTCTTGTCTTATATAC |  |
| HA-P_29_-firefly-R | TCTTTATGTTTTTGGCGTCTTCCATACTTCTACATCCTTCTATAAG |  |
| HA-P_32_-firefly-F | CTACTTCACCTATCCTGCCCTTTTACTTGTTAACAATTGGATAAG |  |
| HA-P_32_-firefly-R | TCTTTATGTTTTTGGCGTCTTCCATTATTAATTAACCTTTTCGGAATAT |  |
| HA-2018β-F | ATCAGTGATAGAGAGAATTCATATGCAAGAGAATAAATCTTTAGTTACCCGT | Used for constructing plasmid pBBR1-kmR-oriT-Ptet-Redαβ2018 |
| 2018β-R | TTAAGCGTTTTCCTCAGCAA |  |
| HA-RBS-2018α-F | TTGCTGAGGAAAACGCTTAA**TAAAGGAGGTTCGAAT**ATGAAAATCGTAAATTTATCTCA |  |
| HA-2018α-R | GGGCTGGGTCTAGAGGATCCTTAAAAATAAAATGACTCTGCTTTTTCAC |  |
| HA-cas9-F | CCCTATCAGTGATAGAGAGAATTCATATGGATAAGAAATACTCAATAGGC | Used for constructing plasmids pBBR1-km^R^-oriT-P_tet_-Cas9 and pBBR1-km^R^-oriT-P_tet_-Redαβ2018-Cas9 |
| cas9-R | TCAGTCACCTCCTAGCTGAC |  |
| HA-sacB-F | GTCAGCTAGGAGGTGACTGAATTTCGCTCGGTACCATCGG |  |
| sacB-R | ACCCATCACATATACCTGCC |  |
| gRNA-F | GGCAGGTATATGTGATGGGT |  |
| gRNA-R | GGGCTGGGTCTAGAGGATCC |  |
| HA-PvuI-2018-F | GTCAGCTAGGAGGTGACTGA*CGATCG*CATTAATTCCTAATTTTTGTTGACAC |  |
| HA-SmaI-2018-R | ACCCATCACATATACCTG*CCCGGG*AAGCACACGGTCACACTGCT |  |
| Cas9-Slp-F | TGGAATTGTGAGCGGATAACAATTTCACACCTATTACTGGTGACTTCTCT | Used for constructing plasmids pBBR1-km^R^-oriT-P_tet_-Redαβ2018-Cas9-*slp*-PvuI with different length HAs |
| Cas9-Slp-R | CGCTTGATAGCTGGGAGAAAC  AGAGAAGTCACCAGTAATAG |  |
| HA-Slp-DownHA-F | CGCTTGATAGCTGGGAGAAACATTGACTGCGCCTAGAGGCA |  |
| Slp-UpHA-R | GTTTCTCCCAGCTATCAAGCG |  |
| PvuI-2018Cas9-200bp-Slp-F | GTCAGCTAGGAGGTGACTGACGCGTTACGCCACGCGTTATTC |  |
| PvuI-2018Cas9-200bp-Slp-R | GTGTCAACAAAAATTAGGAATTAATGCGTGGTCAGCAAGGCGGCTAAT |  |
| PvuI-2018Cas9-100bp-Slp-F | GTCAGCTAGGAGGTGACTGACGTGTGGAAATACAGAACGCTGTA |  |
| PvuI-2018Cas9-100bp-Slp-R | GTGTCAACAAAAATTAGGAATTAATGCGTTGTGAATTTGTTTAAGATATTCGA |  |
| PvuI-2018Cas9-50bp-Slp-F | GTCAGCTAGGAGGTGACTGACGGGAAAATTATATATTTATTACGCTA |  |
| PvuI-2018Cas9-50bp-Slp-R | GTGTCAACAAAAATTAGGAATTAATGCGCGCCTTTATCGCCATAGTGC |  |
| BGC1_4-P_18_-F1 | AGCGGATAACAATTTCACACTTTTACTTGGTAACGCCTCATCGTAAAAATACTGATAGGCAATATCTTGAGATCGAGTACGCGCTAAACC | Used for constructing plasmid pBBR1-km^R^-oriT-P_tet_-Redαβ2018-Cas9-P_18_-BGC1-4 |
| BGC1_4-P_18_-R1 | TGCTATTTCTAGCTCTAAAACAAGGAATCCCAACCAAGTTAAGCGACATTGGTGATCAAAGGAGAGCAAGCGTTTAAGCCTGCTGAAATTTAC |  |
| BGC1_4-P_18_-F2 | GATCGAGTACGCGCTAAACCCGCGAGTATAGTAACAATATTTGTATTCATTTGTGTTCCCTAATCTAGGTTTG |  |
| BGC1_4-P_18_-R2 | TTTAAGCCTGCTGAAATTTACCAAGCGATTGGAGTGAAGCCGTTCACTACCTCGCCTTCGCTAAAGTAAATATCAATCTTTAAATAACTATTGGCT |  |
| CHK-∆Pfl-F | CGGTGTAAATGCCTCTAACT | Used for identifying the genotype of DSM 14401∆*Pfl* |
| CHK-∆Pfl-R | TTGGAAGAAGCGCTCGAGAC |  |

Underlined sequences indicate homology arms.

Italic letters represent restriction enzyme sites.

Bold letters represent ribosome binding sites (RBSs).

**Table S4.** Antibiotic susceptibility test of DSM 14401.

| **Cm 30** | **Spect 100** | **Amp 100** | **Tet 50** | **Genta 10** | **Km 50** | **Km 300** | **Erm 10** |
| --- | --- | --- | --- | --- | --- | --- | --- |
| - | + | + | + | + | ± | - | - |

-: sensitive; +: resistant; ±: sporadic growth.

unit: µg/mL

**Table S5.** Antibiotic susceptibility test of DSM 14401.

| BGC | Type | Size (kb) | Similarity |
| --- | --- | --- | --- |
| BGC1-1 | Thiopeptide | 41.1 | - |
| BGC1-2 | RiPP-like | 10.8 | - |
| BGC1-3 | T2PKS-NRPS | 78.2 | 69% |
| BGC1-4 | NRPS-T1PKS | 70.8 | 6% |
| BGC1-5 | NRPS-betalactone | 122.5 | 60% |
| BGC1-6 | NRPS-lanthipeptide | 59.5 | - |
| BGC1-7 | NRPS | 55.1 | - |
| BGC1-8 | Thiopeptide | 32.9 | 1% |
| BGC1-9 | T1PKS-NRPS | 49.3 | - |
| BGC2-1 | NRPS | 84.5 | 1% |
| BGC2-2 | RiPP-like | 8.1 | - |
| BGC2-3 | RiPP-like | 11.0 | - |
| BGC2-4 | Acyl-amino-acids | 60.9 | - |

**Table S6.** The ^1^H (600 MHz) and ^13^C NMR (150 MHz) Data of flavipulchrin A in CD_3_OH

|  | no | flavipulchrin A | |
| --- | --- | --- | --- |
|  |  | *δ*_C_, Type | *δ*_H_, Mult. (*J* in Hz) |
| 3-Amino-dodecanoic acid | 1  2a  2b  3  4  5a  5b  6  7  8  9  10  11  12  3-NH | 175.0, C  44.1, CH_2_  48.9, CH  36.4, CH_2_  27.2, CH_2_  30.3, CH_2_  30.6, CH_2_  30.6, CH_2_  30.3, CH_2_  32.9, CH_2_  23.6, CH_2_  14.3, CH_3_ | 2.45, dd (1.7, 13.9)  2.22, dd (10.4, 13.9)  4.15, m  1.50, m*^a^*  1.37, m  1.29, m*^a^*  1.29, m*^a^*  1.29, m*^a^*  1.29, m*^a^*  1.29, m*^a^*  1.29, m*^a^*  1.29, m*^a^*  0.89, t (6.9)  8.06, d (8.5) |
| Gly | 1  2a  2b  2-NH | 171.8, C  44.2, CH_2_ | 3.95, dd (6.8, 17.3)  3.68, dd (4.7, 17.3)  8.47, t (5.8) |
| Asp | 1  2  3a  3b  4  2-NH | 174.8, C  52.1, CH  39.0, CH_2_  178.5, C | 4.76, m  3.30, m*^a^*  2.67, dd (3.7, 15.1)  8.45, d (7.6) |
| OH-Asn | 1  2  3  4  2-NH  4-NH | 170.8, C  59.5, CH  72.4, CH  175.7, C | 4.56, m  4.45, m*^a^*  8.34, brs  7.58, 7.27, brs |
| Asn | 1  2  3a  3b  4  2-NH  4-NH | 174.5, C  54.1, CH  37.5, CH_2_  174.3, C | 4.60, m  3.07, dd (10.6, 14.5)  2.63, dd (3.3, 14.5)  8.45, d (7.6)  7.88, 6.98, brs |
| Thr | 1  2  3  4  2-NH | 174.0, C |  |
|  |  | 60.3, CH | 4.43, m |
|  |  | 69.8, CH | 4.27, m |
|  |  | 19.8, CH_3_ | 1.19, d (6.4)  7.58, brs |
| Leu | 1  2  3a  3b  4  5  6  2-NH | 175.1, C |  |
|  |  | 54.8, CH | 4.23, m |
|  |  | 40.6, CH_2_  25.6, CH  23.9, CH_3_  21.0, CH_3_ | 1.79, m*^a^*  1.50, m*^a^*  1.79, m*^a^*  0.95, d (6.5)  0.85, d (6.5)  8.05, brs |
| Arg | 1  2  3  4  5  6  2-NH  5-NH | 173.3, C  53.8, CH  31.6, CH_2_  26.0, CH_2_  42.2, CH_2_  158.7, C | 4.45, m*^a^*  1.73, m  1.62, m  3.15, m  7.64, d (8.3)  7.32, brs |

*^a^* overlapped

**Table S7.** The ^1^H (600 MHz) and ^13^C NMR (150 MHz) Data of flavipulchrin B in CD_3_OH

|  | no | flavipulchrin B | |
| --- | --- | --- | --- |
|  |  | *δ*_C_, Type | *δ*_H_, Mult. (*J* in Hz) |
| 3-Amino-undecanoic acid | 1  2a  2b  3  4  5a  5b  6  7  8  9  10  11  3-NH | 175.0, C  44.1, CH_2_  48.8, CH  36.4, CH_2_  27.2, CH_2_  30.3, CH_2_  30.5, CH_2_  30.3, CH_2_  32.9, CH_2_  23.6, CH_2_  14.3, CH_3_ | 2.44, dd (1.8, 14.0)  2.22, dd (9.6, 14.0)  4.15, m  1.51, m*^a^*  1.37, m  1.29, m*^a^*  1.29, m*^a^*  1.29, m*^a^*  1.29, m*^a^*  1.29, m*^a^*  1.29, m*^a^*  0.89, t (6.7)  8.07, d (8.4) |
| Gly | 1  2a  2b  2-NH | 171.8, C  44.2, CH_2_ | 3.94, dd (6.8, 17.2)  3.68, dd (4.7, 17.2)  8.45, t (5.9) |
| Asp | 1  2  3a  3b  4  2-NH | 174.7, C  52.1, CH  38.8, CH_2_  178.4, C | 4.76, m  3.30, m*^a^*  2.66, m*^a^*  8.45, d (7.9) |
| OH-Asn | 1  2  3  4  2-NH  4-NH | 170.7, C  59.5, CH  72.4, CH  175.7, C | 4.56, m  4.45, m*^a^*  8.29, brs  7.58, 7.24, brs |
| Asn | 1  2  3a  3b  4  2-NH  4-NH | 174.5, C  54.1, CH  37.5, CH_2_  174.3, C | 4.59, m  3.06, dd (10.6, 14.9)  2.66, m*^a^*  8.43, d (6.2)  7.87, 6.95, brs |
| Thr | 1  2  3  4  2-NH | 174.0, C | 4.43, m  4.27, m  1.18, d (6.4)  7.58, brs |
|  |  | 60.3, CH |  |
|  |  | 69.8, CH |  |
|  |  | 19.8, CH_3_ |  |
| Leu | 1  2  3a  3b  4  5  6  2-NH | 175.1, C |  |
|  |  | 54.8, CH | 4.23, m |
|  |  | 40.6, CH_2_  25.6, CH  23.9, CH_3_  21.0, CH_3_ | 1.81, m*^a^*  1.51, m*^a^*  1.81, m*^a^*  0.95, d (6.4)  0.85, d (6.5)  8.05, brs |
| Arg | 1  2  3  4  5  6  2-NH  5-NH | 173.3, C  53.8, CH  31.5, CH_2_  25.9, CH_2_  42.2, CH_2_  158.7, C | 4.45, m*^a^*  1.73, m  1.61, m  3.17, m  7.64, d (8.3)  7.32, brs |

*^a^* overlapped


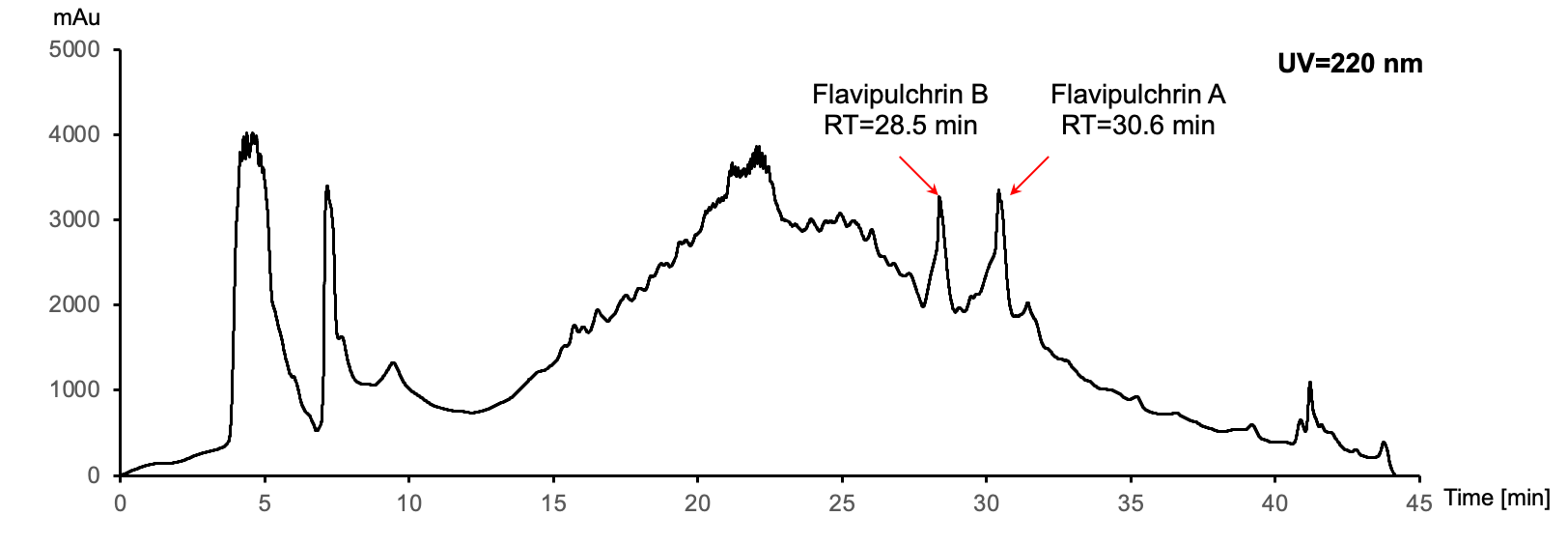


**Figure S1.** Semipreparative HPLC (UV=220 nm) chromatogram during purification of flavipulchrin A and B.


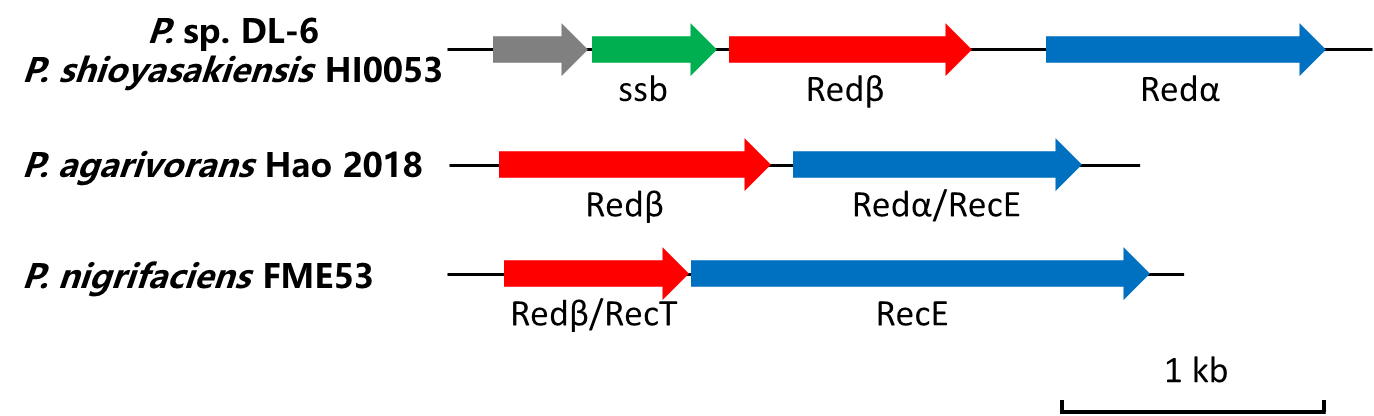


**Figure S2.** Three Red/ET systems across four *Pseudoalteromonas* strains


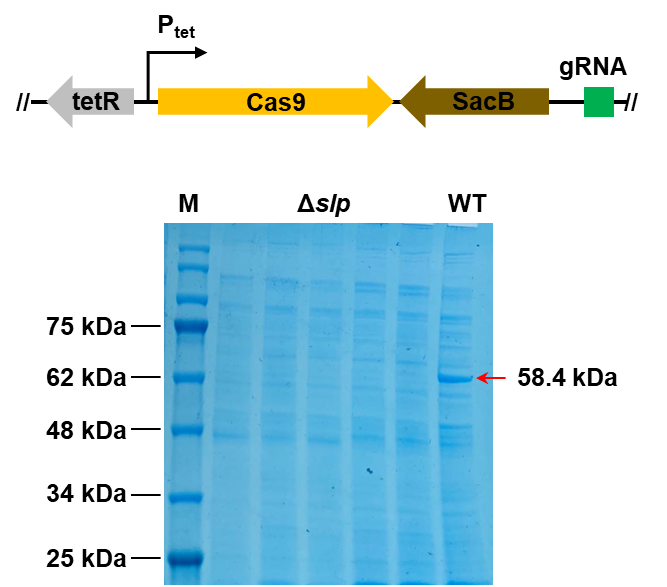


**Figure S3.** CRISPR/Cas9 plasmid architecture and editing validation in DSM 14401


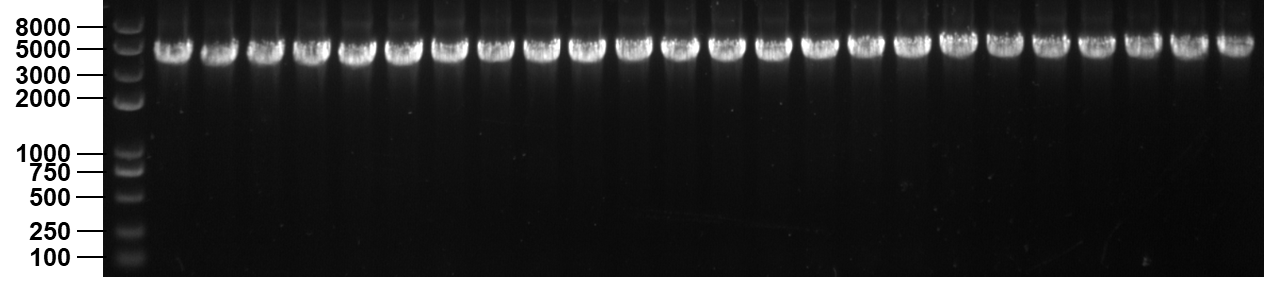


**Figure S4.** Detection of Cas9 integrity via PCR


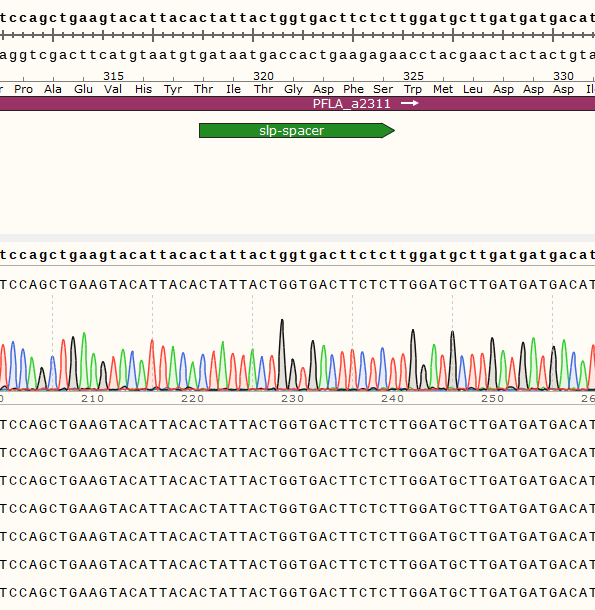


**Figure S5.** Protospacer sequence analysis of CRISPR escape mutants


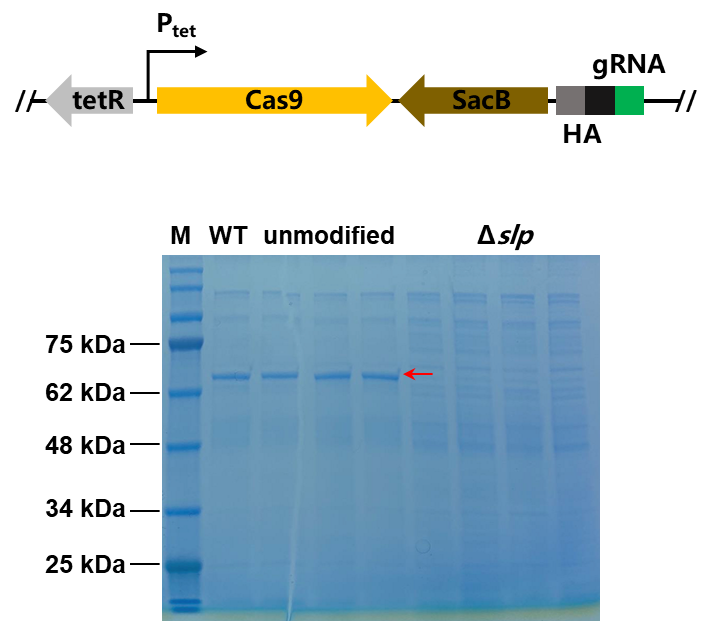


**Figure S6.** The plasmid map of pBBR1-km-P_tet_-Cas9-SacB-200bp-SLP and its editing validation in DSM 14401


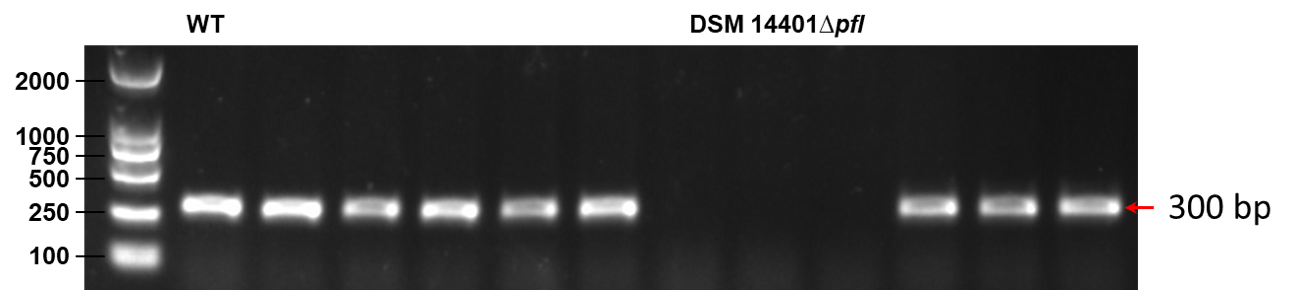


**Figure S7.** Fragment deletion mediated by CRISPR/Cas9 in DSM 14401


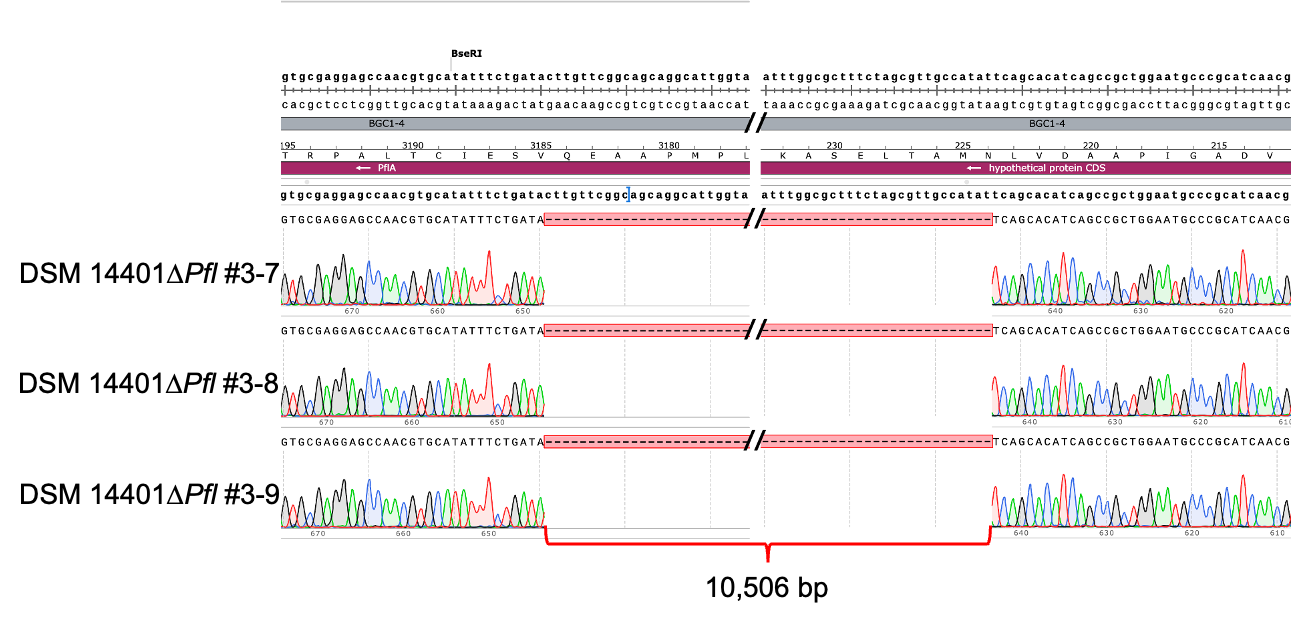


**Figure S8.** Sanger sequencing of DSM 14401∆*Pfl* mutants.

**
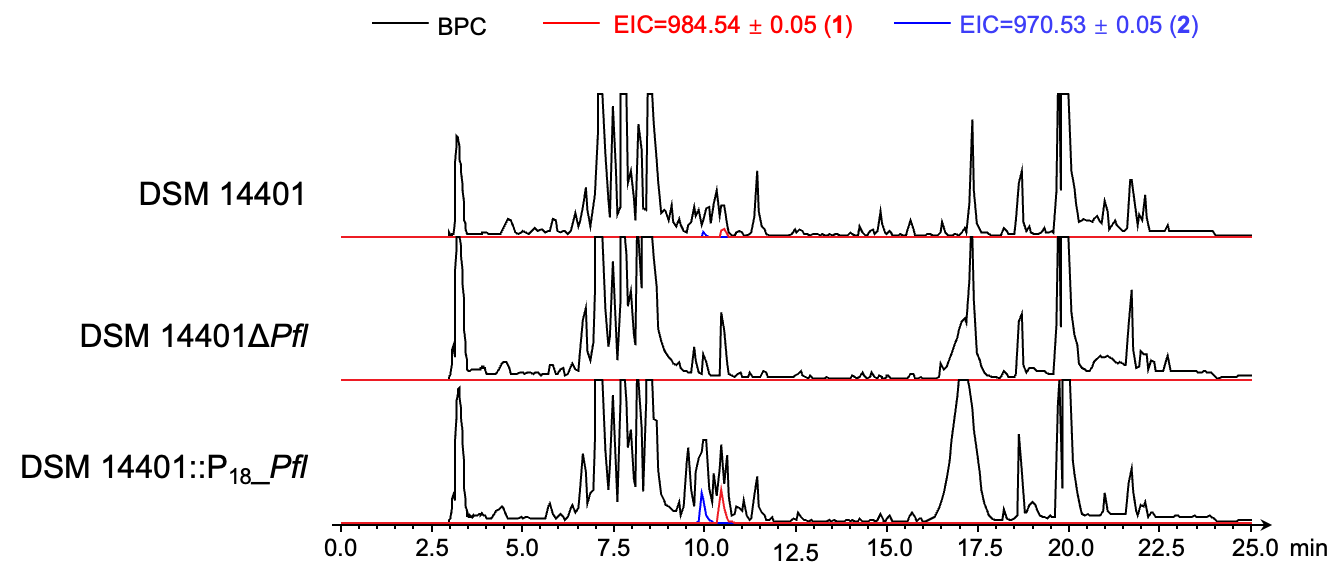
**

**Figure S9.** LC-MS analysis of wild type *P. flavipulchra* DSM 14401 and its BGC1-4 inactivation and activation mutants, black lines represent BPCs (Base Peak Chromatograms), red or blue lines represents EICs (Extracted Ion Chromatograms).

**
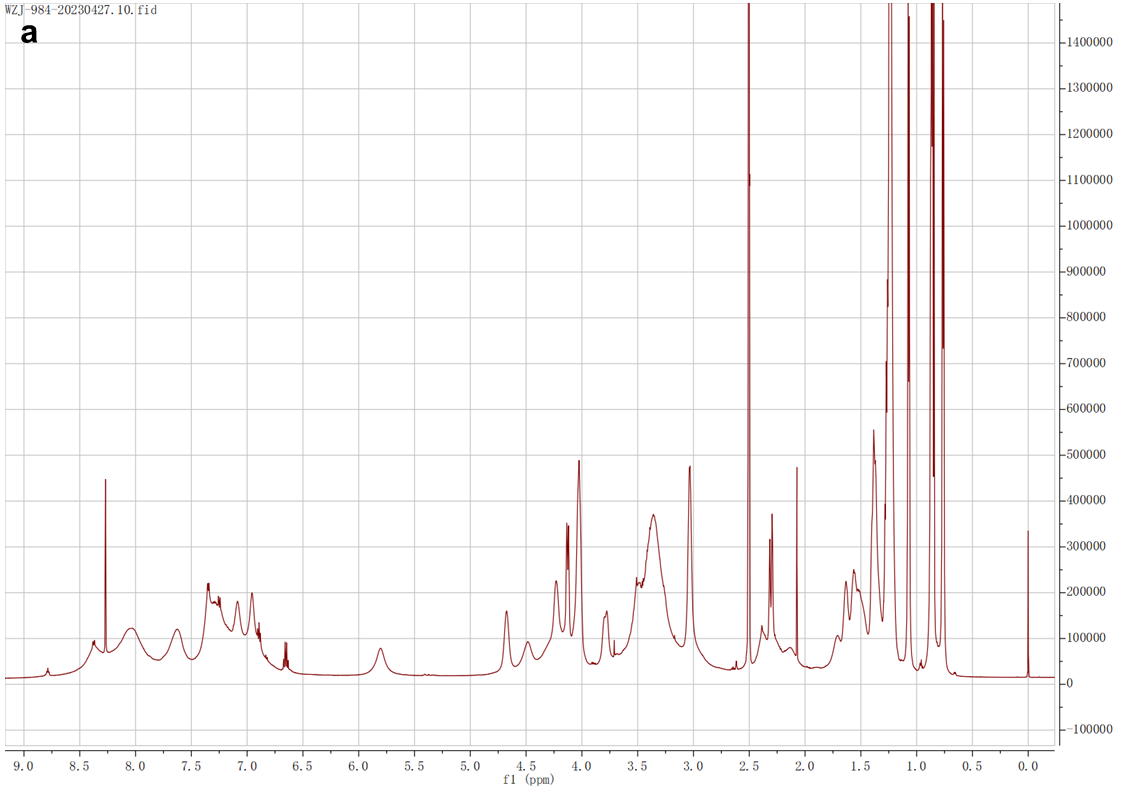
**

**
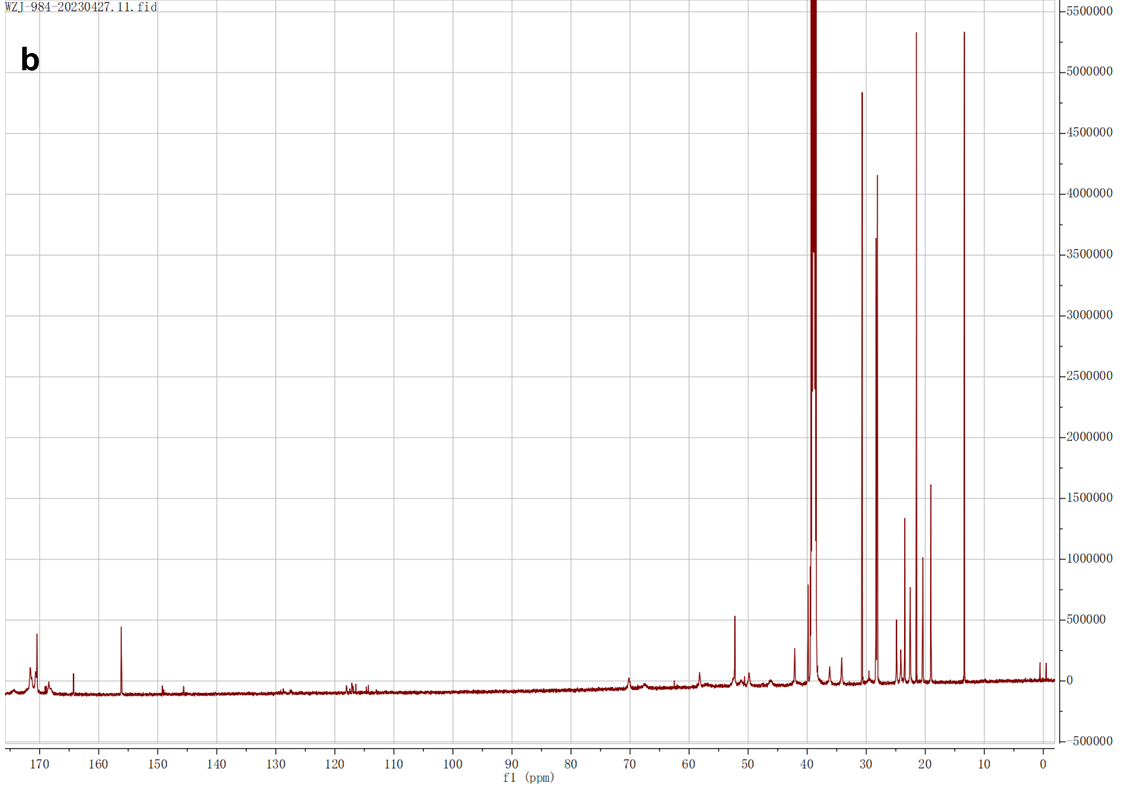
**

**Figure S10.** NMR spectra of flavipulchrin A (**1**) in DMSO-*d*6. (**a**) ^1^H NMR spectrum; (**b**) ^13^C NMR spectrum;


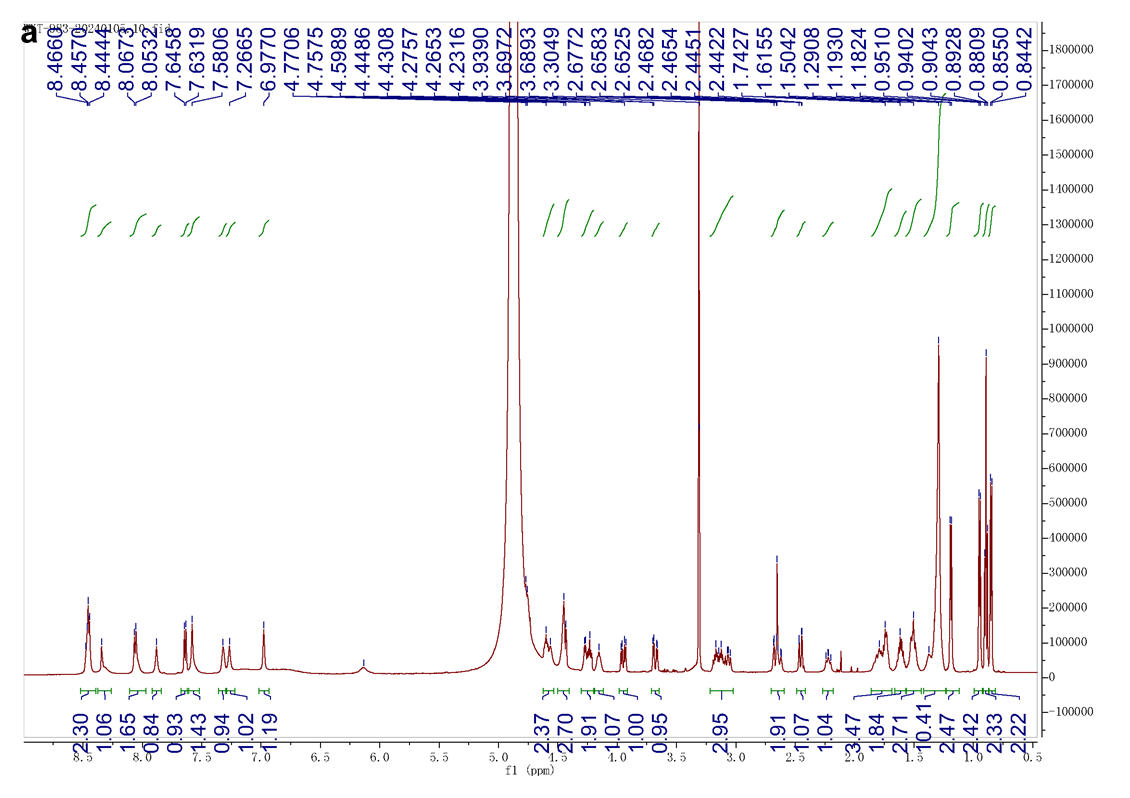


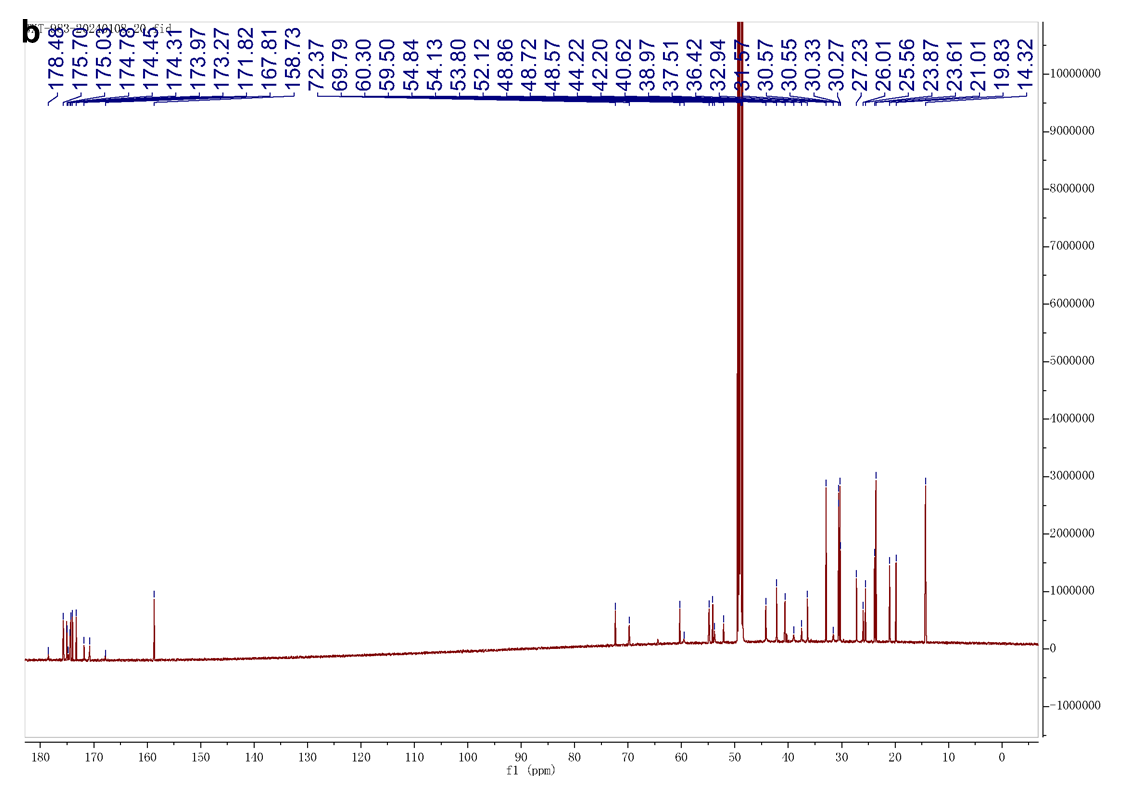


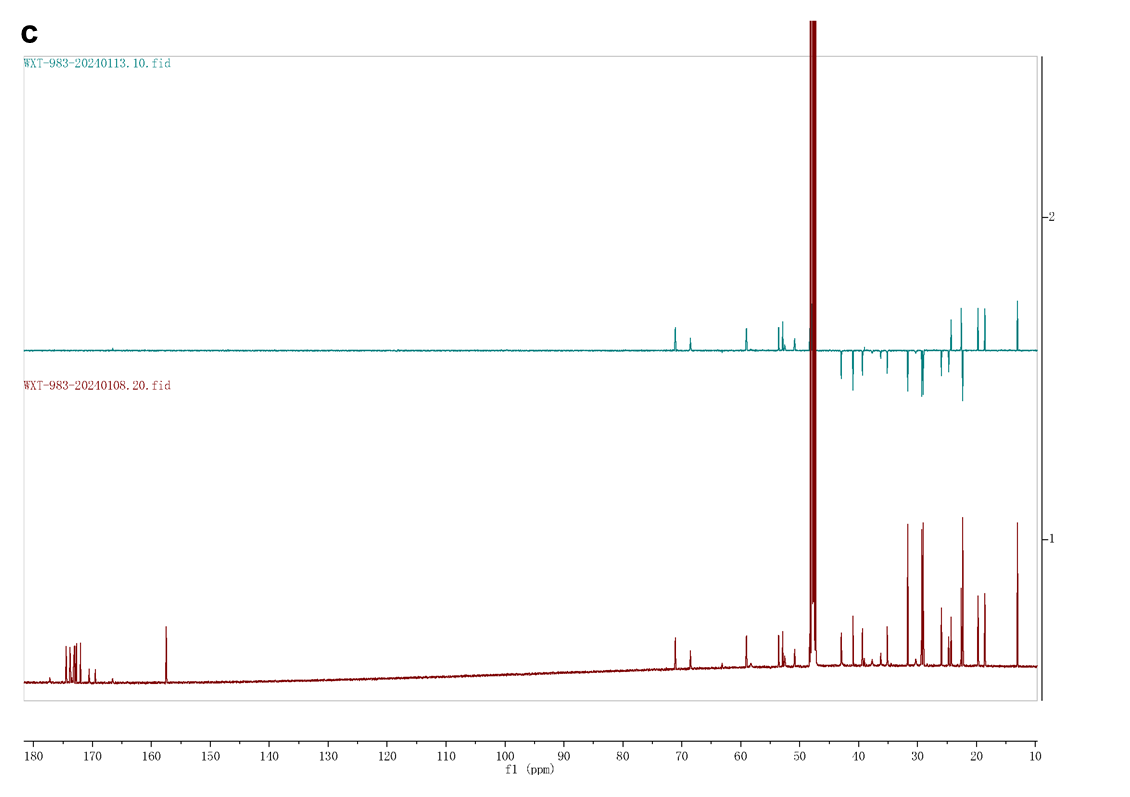


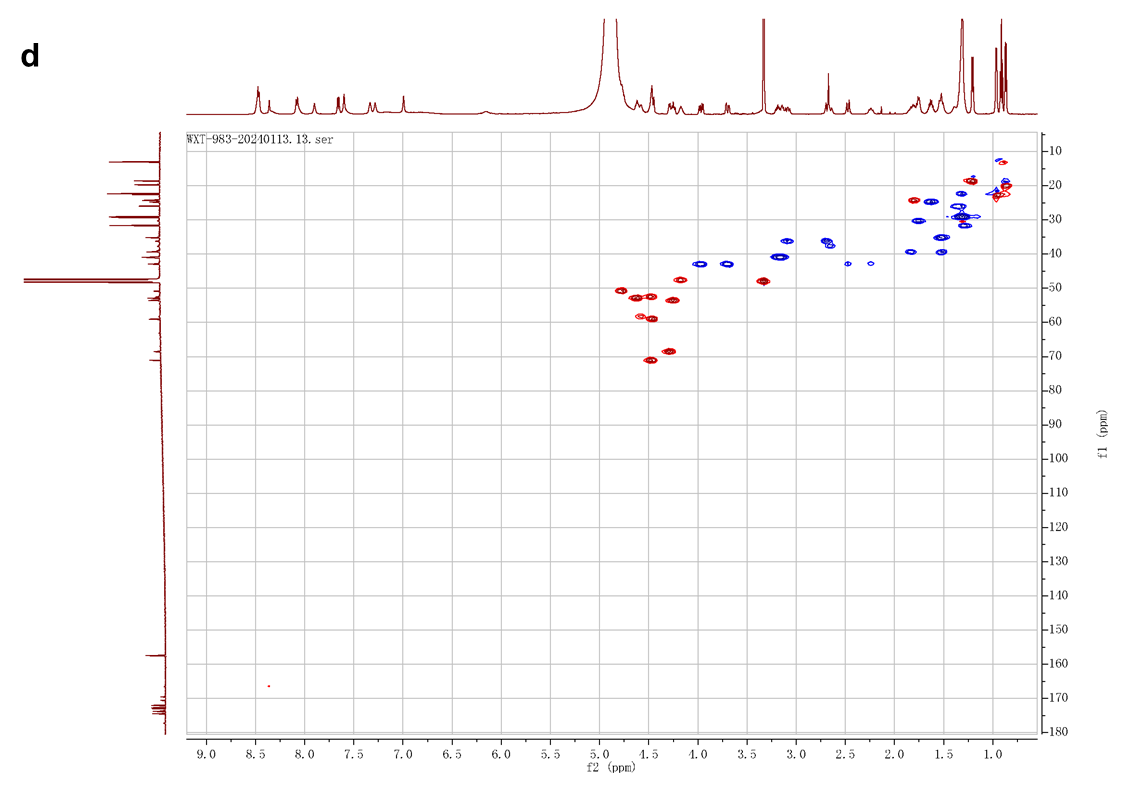


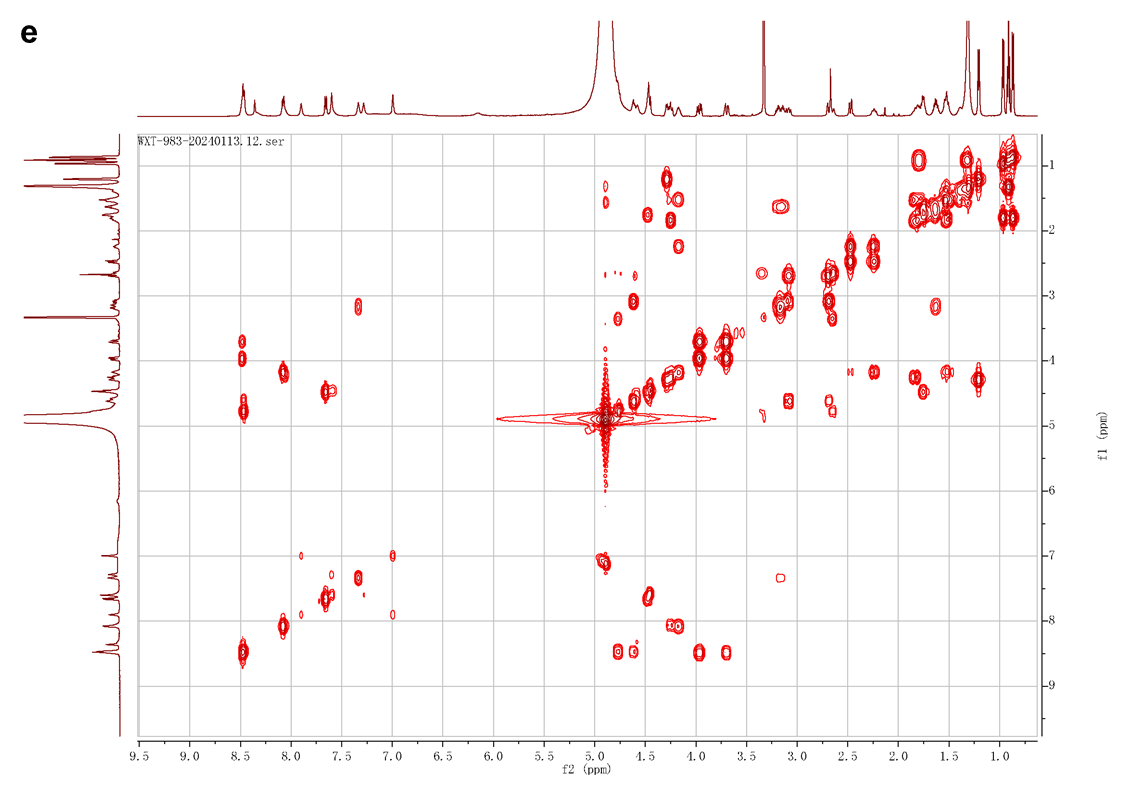


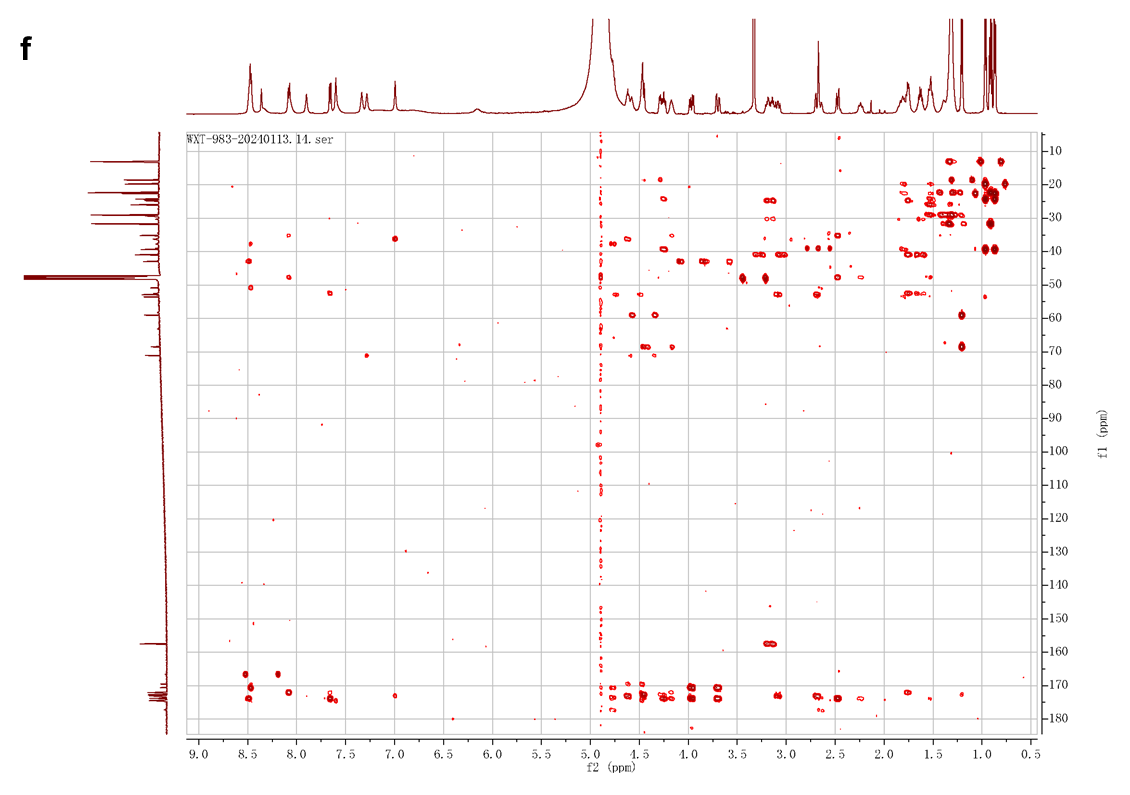


**Figure S11.** NMR spectra of flavipulchrin A (**1**) in MeOH-*d*3. (**a**) ^1^H NMR spectrum; (**b**) ^13^C NMR spectrum; (**c**) DEPT spectrum; (**d**) HSQC spectrum; (**e**) ^1^H-^1^H COSY spectrum; (**f**) HMBC spectrum.

**
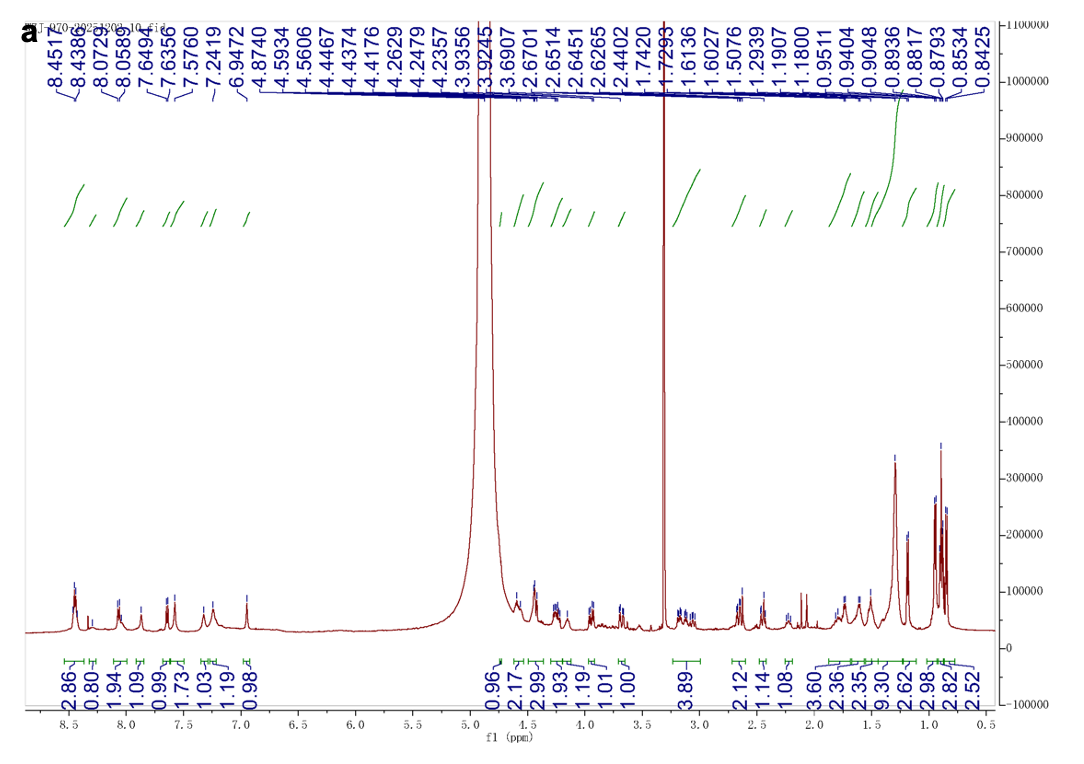
**

**
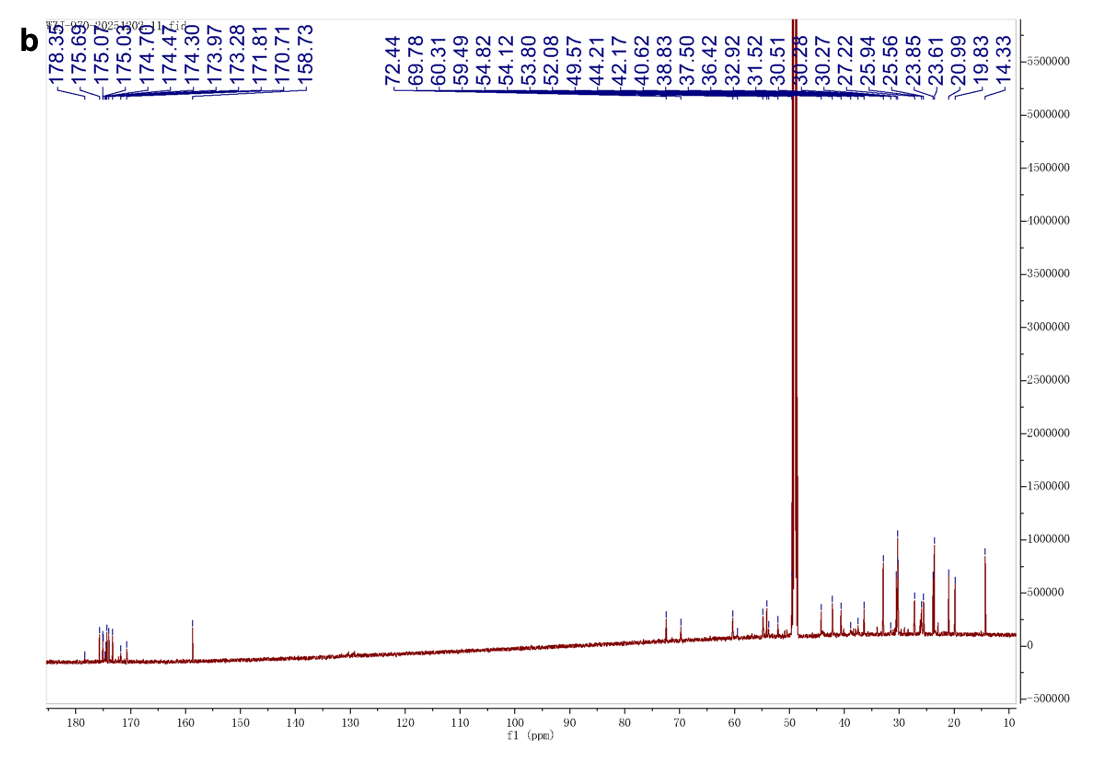
**

**
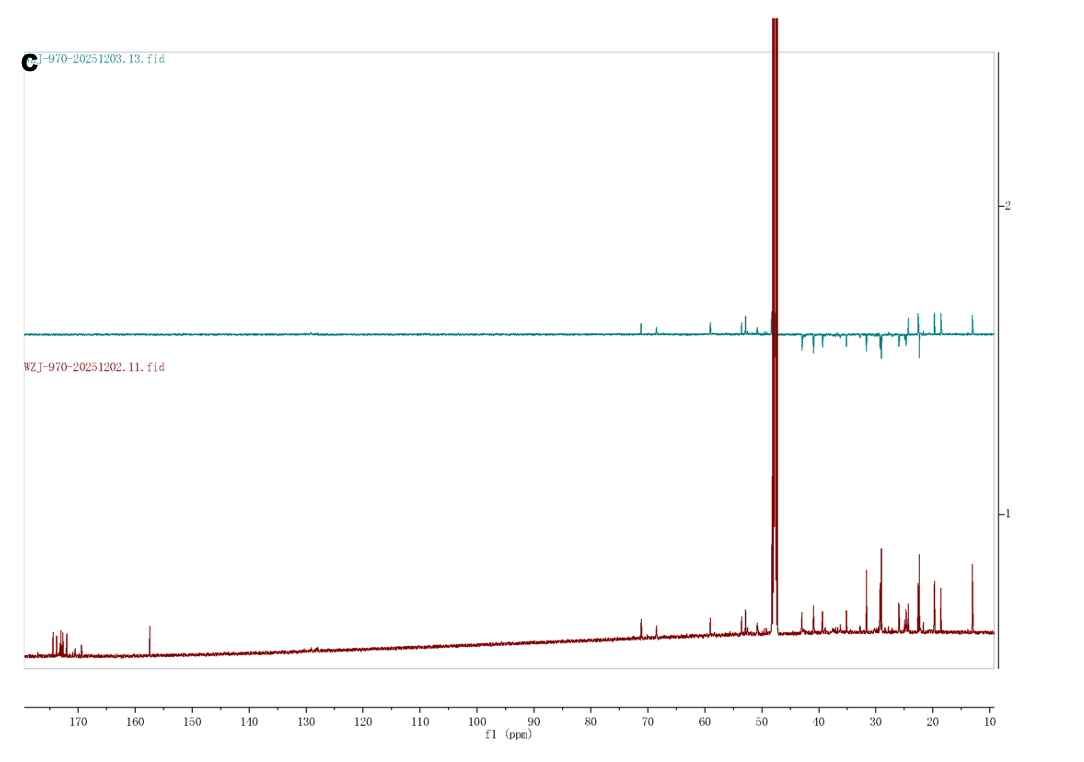
**

**
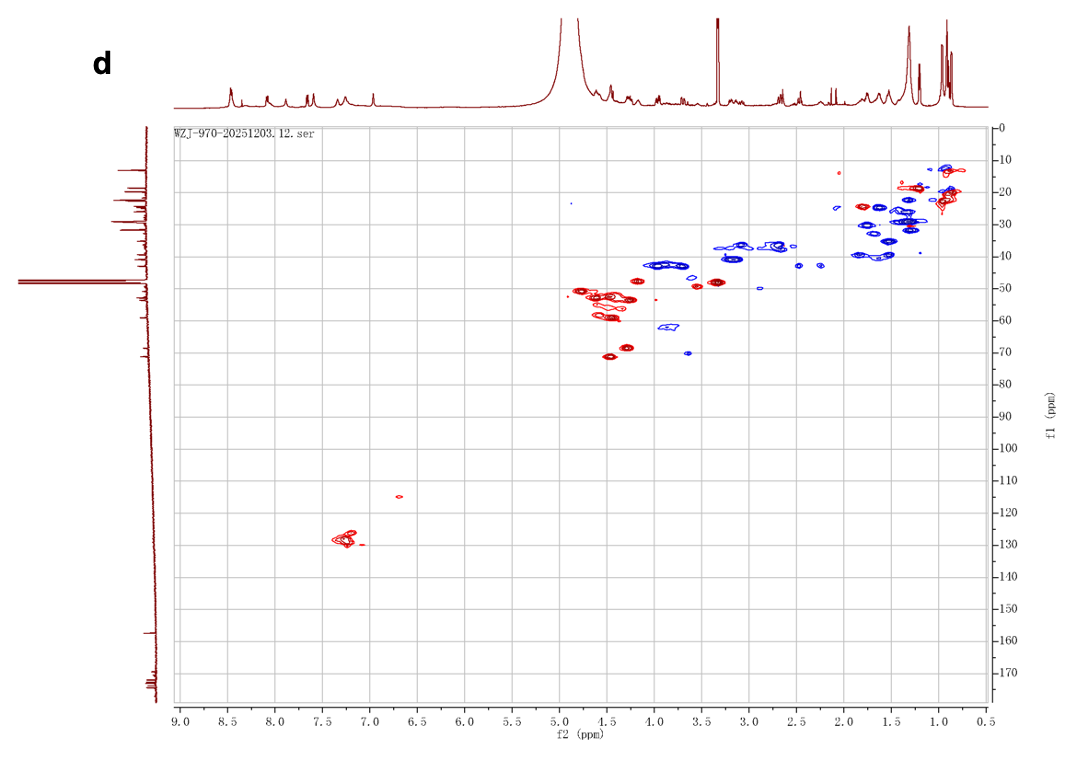
**

**
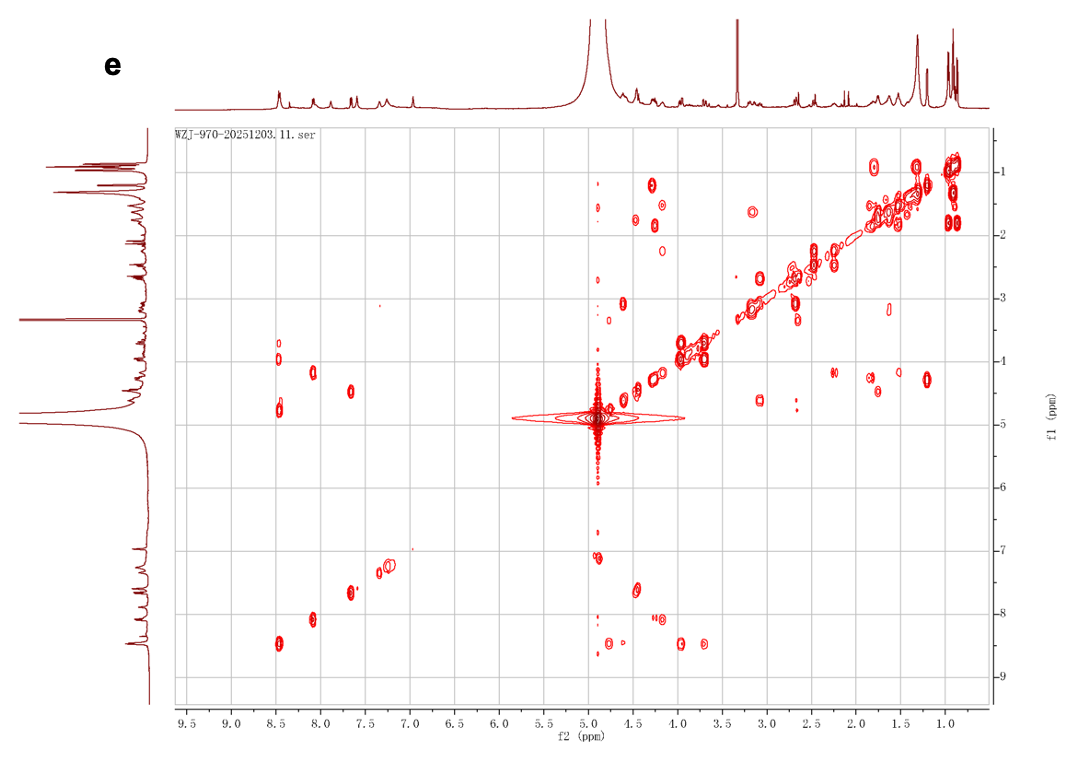
**

**
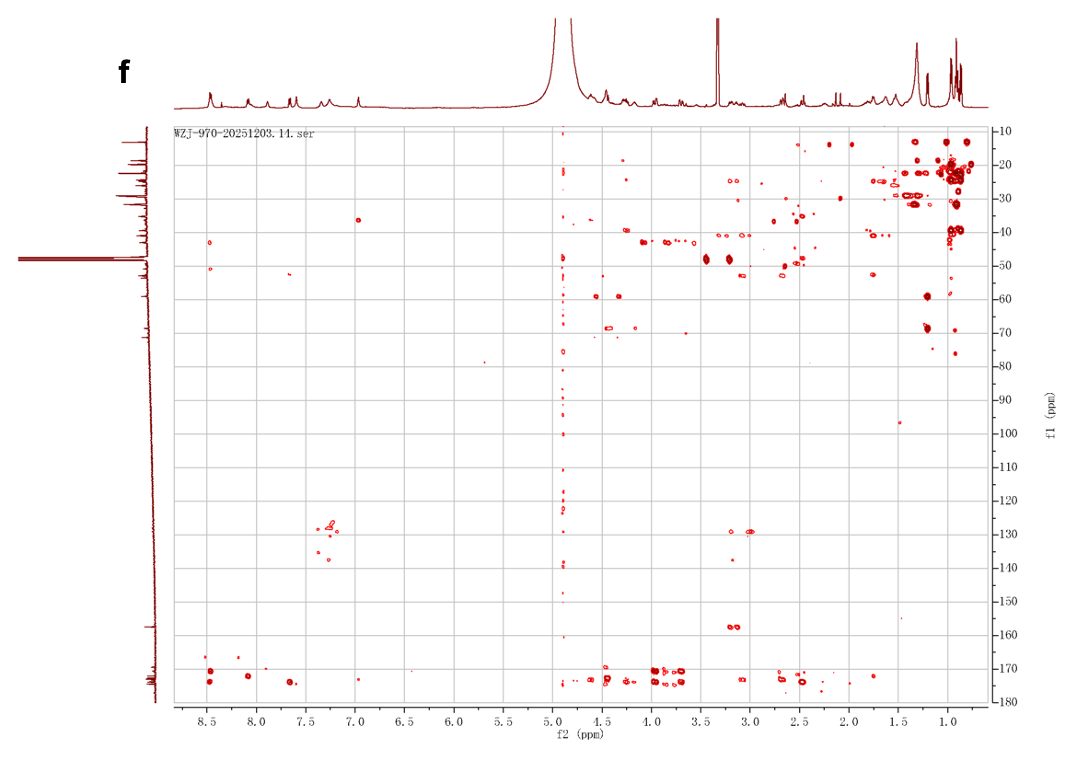
**

**Figure S12.** NMR spectra of flavipulchrin B (**2**) in MeOH-*d*3. (**a**) ^1^H NMR spectrum; (**b**) ^13^C NMR spectrum; (**c**) DEPT spectrum; (**d**) HSQC spectrum; (**e**) ^1^H-^1^H COSY spectrum; (**f**) HMBC spectrum.


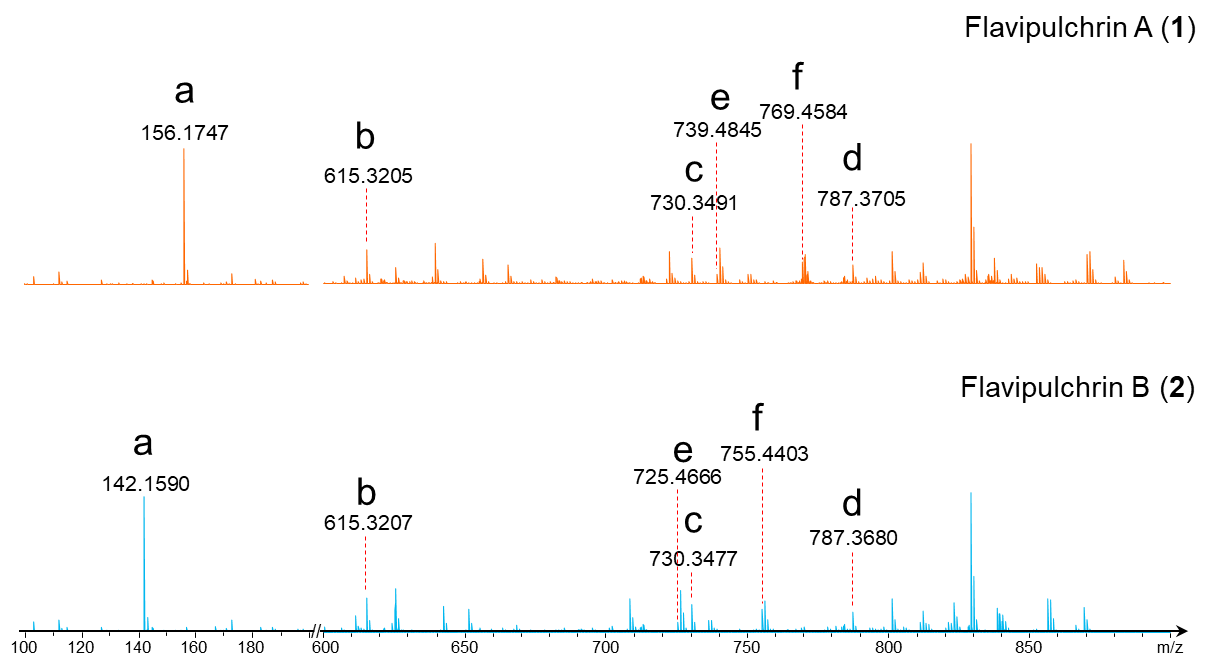

**b**

**a**

**c**

**d**

**e**

**f**

**Figure S13.** Major characteristic fragmentation ions of flavipulchrin A (**1**) and B (**2**) in MS/MS spectra.


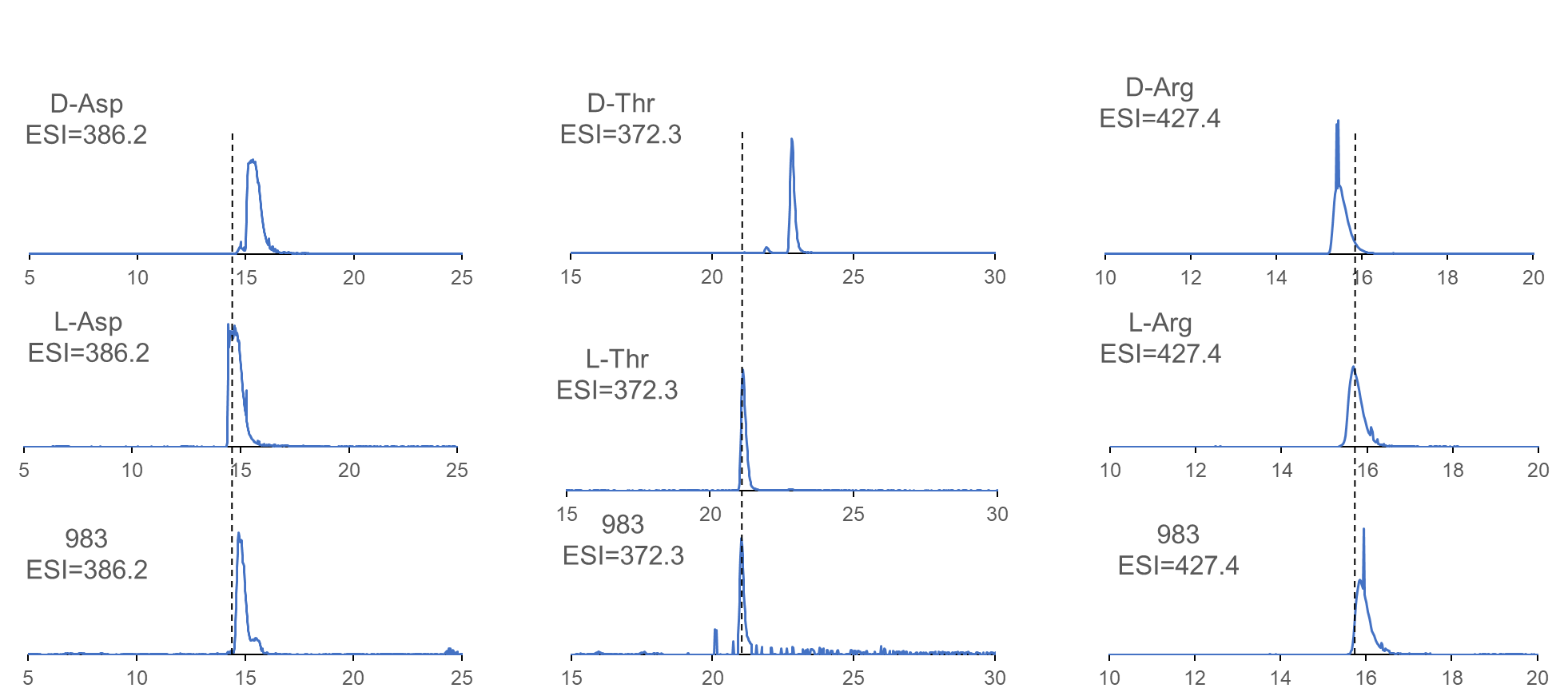


**Figure S14.** LC-MS analysis of the stereo configuration of Asp, Thr and Arg.


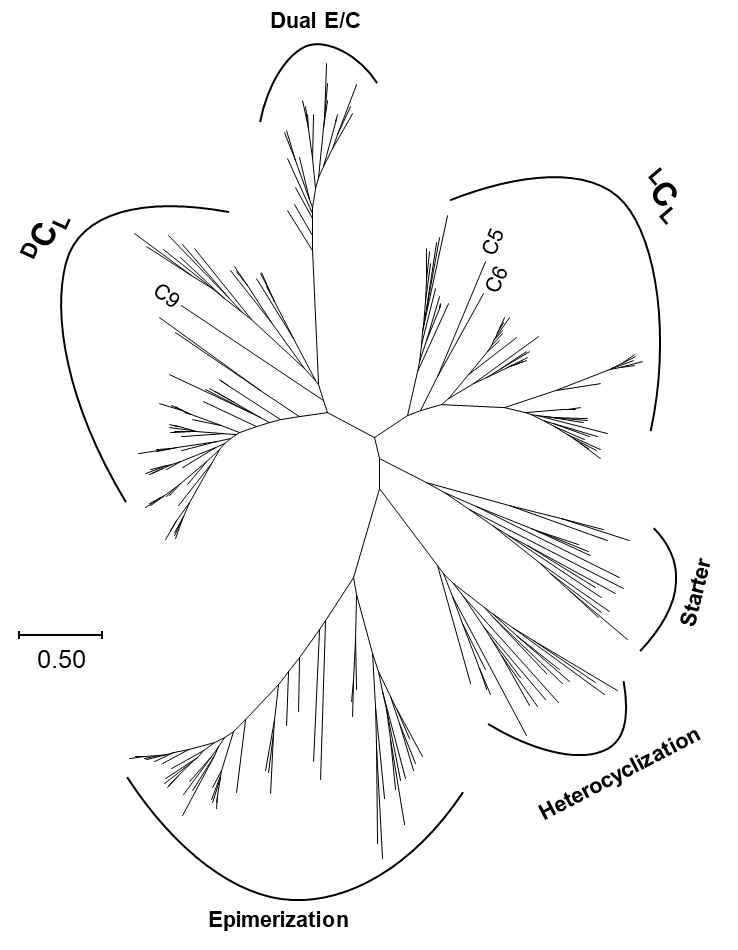


**Figure S15.** Phylogenetic tree of C domain. The Maximum Likelihood (ML) tree was reconstructed using MEGA 12.0, employing the JTT model of amino acid substitution.

**
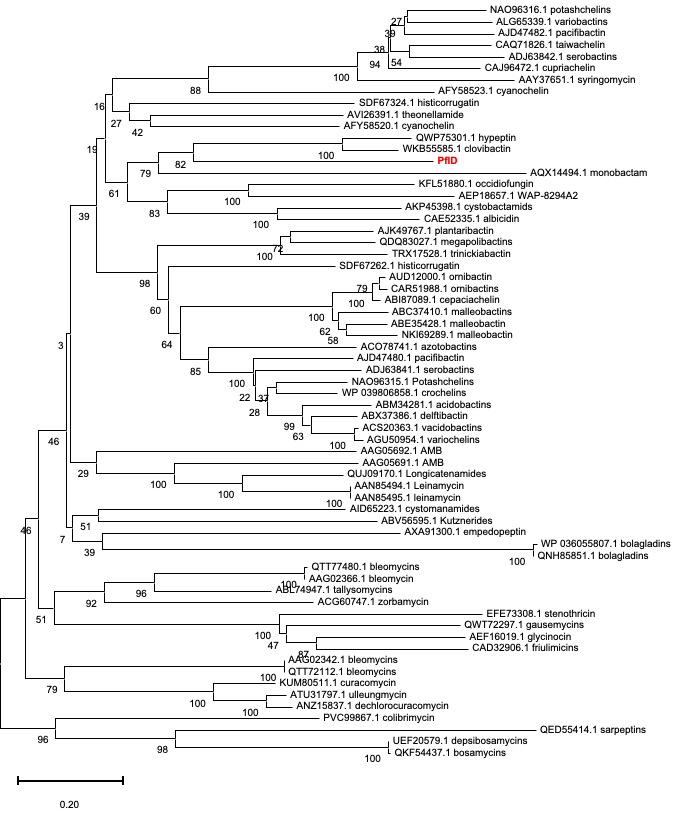
**

**Figure S16.** Phylogenetic tree of PflD. The Neighbor-Joining (NJ) tree was reconstructed using MEGA 12.0, employing the JTT model of amino acid substitution.

**Reference**

(1) Dehio, C.; Meyer, M. Maintenance of broad-host-range incompatibility group P and group Q plasmids and transposition of Tn5 in Bartonella henselae following conjugal plasmid transfer from Escherichia coli. *Journal of bacteriology* **1997**, *179* (2), 538-540. DOI: 10.1128/jb.179.2.538-540.1997.
